# Kremen2 drives the progression of non-small cell lung cancer by preventing SOCS3-mediated degradation of EGFR

**DOI:** 10.1186/s13046-023-02692-3

**Published:** 2023-06-03

**Authors:** Yuxiao Sun, Yu Gao, Mingxin Dong, Jiuzhen Li, Xin Li, Ningning He, Huijuan Song, Manman Zhang, Kaihua Ji, Jinhan Wang, Yeqing Gu, Yan Wang, Liqing Du, Yang Liu, Qin Wang, Hezheng Zhai, Daqiang Sun, Qiang Liu, Chang Xu

**Affiliations:** 1 Tianjin Key Laboratory of Radiation Medicine and Molecular Nuclear Medicine, Institute of Radiation Medicine, Chinese Academy of Medical Science and Peking Union Medical College, Tianjin, 300192 China; 2 Graduate School, Tianjin Medical University, Tianjin, 300070 China; 3 Department of Thoracic Surgery, Tianjin Chest Hospital of Tianjin University, Tianjin, 300222 China; 4 School of Precision Instruments and OPTO-Electronics Engineering, Tianjin University, Tianjin, 300072 China

**Keywords:** Kremen2, Non-small cell lung cancer, EGFR, SOCS3, Ubiquitination

## Abstract

**Background:**

The transmembrane receptor Kremen2 has been reported to participate in the tumorigenesis and metastasis of gastric cancer. However, the role of Kremen2 in non-small cell lung cancer (NSCLC) and the underlying mechanism remain unclear. This study aimed to explore the biological function and regulatory mechanism of Kremen2 in NSCLC.

**Methods:**

The correlation between Kremen2 expression and NSCLC was assessed by analyzing the public database and clinical tissue samples. Colony formation and EdU assays were performed to examine cell proliferation. Transwell and wound healing assays were used to observe cell migration ability. Tumor-bearing nude mice and metastatic tumor models were used to detect the in vivo tumorigenic and metastatic abilities of the NSCLC cells. An immunohistochemical assay was used to detect the expression of proliferation-related proteins in tissues. Western blot, immunoprecipitation and immunofluorescence were conducted to elucidate the Kremen2 regulatory mechanisms in NSCLC.

**Results:**

Kremen2 was highly expressed in tumor tissues from NSCLC patients and was positively correlated with a poor patient prognosis. Knockout or knockdown of Kremen2 inhibited cell proliferation and migration ability of NSCLC cells. In vivo knockdown of Kremen2 inhibited the tumorigenicity and number of metastatic nodules of NSCLC cells in nude mice. Mechanistically, Kremen2 interacted with suppressor of cytokine signaling 3 (SOCS3) to maintain the epidermal growth factor receptor (EGFR) protein levels by preventing SOCS3-mediated ubiquitination and degradation of EGFR, which, in turn, promoted activation of the PI3K-AKT and JAK2-STAT3 signaling pathways.

**Conclusions:**

Our study identified Kremen2 as a candidate oncogene in NSCLC and may provide a potential target for NSCLC treatment.

**Supplementary Information:**

The online version contains supplementary material available at 10.1186/s13046-023-02692-3.

## Background

Lung cancer is a common malignancy and the leading cause of cancer-related mortality worldwide [1]. Non-small cell lung cancer (NSCLC) accounts for approximately 85% of lung cancers [2]. The clinical prognosis of NSCLC patients is poor with an overall 5-year survival rate of 15% [3, 4]. Fortunately, the advent of targeted therapies has led to significant advances in the treatment of NSCLC. However, despite the initial high response rates, patients on NSCLC-targeted drugs inevitably become resistant to treatment. Therefore, new targets based on biomarkers are needed.

Kringle containing transmembrane protein 2 (Kremen2) is a single-transmembrane-domain protein composed of 473 amino acids. Kremen2 is an important regulator in the classical Wnt signaling pathway. Kremen2 functionally cooperates with dickkopf homolog 1 (DKK1) to block Wnt/β-catenin signaling. Early studies on Kremen2 focused on its effects on embryonic development and bone formation [5, 6], while the important role of Kremen2 in cancers has been slowly discovered in recent years. Multiple myeloma cells promote the expression of Kremen2 in stromal cells (SCs), thereby inhibiting Wnt signaling in SCs [7]. Moreover, Kremen2 is highly expressed in some colorectal cancer tissues and may be involved in the Wnt non-canonical pathway, but the specific mechanism has not been further investigated [8]. Sumia et al. reported that more than 70% of tissue samples from 18 different cancer types show significantly higher Kremen2 expression levels in tumor tissues than in paired normal tissues according to The Cancer Genome Atlas (TCGA) analysis, particularly in squamous lung cancer where Kremen2 expression levels are more than tenfold higher [9]. In addition, Kremen2 expression is positively associated with the development of gastric cancer [10]. However, its biological function and clinical significance in NSCLC remain unclear.

Epidermal growth factor receptor (EGFR) is a receptor tyrosine kinase (RTK) that is a recognized and effective target for treating NSCLC. Abnormal activation of the EGFR was believed to be a major cause of malignant transformation and cancer metastasis [11]. Oncogenic activation of the EGFR can be induced by a variety of mechanisms, such as gene mutation, transcriptional overexpression, and defective EGFR degradation [12, 13]. Several studies have shown that the stability of the EGFR is an important determinant in regulating the progression of lung cancer and that dysregulation of EGFR degradation accelerates tumorigenesis and progression [14, 15]. Indeed, changes in the EGFR endocytic and recycling pathways in tumor cells may derail the spatial distribution of the EGFR, leading to a persistent oncogenic signaling output [16]. Therefore, promoting the degradation of EGFR is an alternative strategy for targeting EGFR-related cancers. The E3 ubiquitin ligase c-Casitas B-lineage lymphoma (c-CBL) is the most studied ubiquitin ligase in RTK regulation, which includes the degradation of the EGFR [17]. Another class of proteins includes the suppressor of cytokine signaling (SOCS) proteins, which play an important role in regulating of various RTK signaling pathways [18].

The SOCS family of proteins are well-known negative regulators of cytokine receptor signaling. SOCS3 is a member of the SOCS family of proteins and has been suggested to function as a tumor suppressor by inhibiting the JAK/STAT signaling pathway [19]. It has been demonstrated that the interaction between SOCS3 and the EGFR COOH-terminal domain inhibits STAT3 activation by EGFR [20]. Furthermore, the role of SOCS3 in cytokine and growth hormone signaling has been extensively studied. SOCS3 promotes insulin resistance by targeting the insulin receptor substrates (IRS1 and IRS2) for degradation [21]. SOCS3 exhibits different profiles in different cancers. For example, higher SOCS3 expression levels are associated with a better clinical prognosis in breast cancer [22]; loss of SOCS3 function promotes abnormal cell proliferation and migration in hepatocellular carcinoma and colorectal carcinoma [23, 24]; deleting SOCS3 disrupts the formation of the E3 ligase complex and drives integrin β1-mediated small-cell lung cancer metastasis [25]. In previous studies, SOCS3 has been primarily evaluated in NSCLC based on its methylation silencing leading to loss of function [26, 27].

In the present study, we found that Kremen2 was highly expressed in NSCLC and was negatively associated with patient survival. We provide in vitro and in vivo evidence to support the tumor-promoting effect of Kremen2 in NSCLC progression and metastasis. More importantly, we identified that the elevated Kremen2 expression was associated with increased stability of the EGFR. Kremen2 interacted with SOCS3 and SOCS3 destabilized the EGFR through the ubiquitin protein degradation pathway. Our results suggest that Kremen2 may prevent the EGFR degradation and play an important role in tumor progression and metastasis of NSCLC.

## Materials and methods

### Clinical tissue samples

Tumor samples and paired normal samples were collected from NSCLC patients who underwent surgical resection at the Tianjin Chest Hospital. These NSCLC tissue samples were immediately frozen in liquid nitrogen and stored at − 80 °C for subsequent experiments. The cases were collected based on a clear pathological diagnosis and patient consent, and this study was approved by the Internal Review and Science Committee of the Tianjin Chest Hospital.

### Cell culture and reagents

IMR90, MRC5, H1299, A549, H460, H1703, H4006, H358, and HEK293T cells were purchased from the ATCC (ATCC, Manassas, VA, USA). IMR90, MRC5, and HEK293T cells were cultured in DMEM with 10% fetal bovine serum (FBS) (Hakata, Shanghai, China). H1299, A549, H460, H1703, H4006, and H358 cells were cultured in RPMI-1640 with 10% FBS. The cells were cultured in a standard humidified incubator at 37 °C in a 5% CO_2_ air atmosphere. The cells were treated with 50 ng/mL of the EGF protein (HY-P7109, MCE) for 15 min to activate EGFR signaling.

### Generation of Kremen2 and SOCS3 knockout cells

Three CRISPR guide RNAs (gRNAs) of Kremen2 were designed by Shanghai Genechem Co., Ltd. (Shanghai, China) and cloned into the lentiviral vector. The gRNA sequences were: sgKremen2_1: 5′- CGTGCAGCCGTGGTGCTACG-3′; sgKremen2_2: 5′­CGCGGCCACCAGAACCGCAC-3′; sgKremen2_3: 5′-GTCTCAGCCACGTAGCACCA-3′. A549 cells were infected with lentivirus and then selected in culture medium containing 2 μg/ml puromycin (InvivoGen, San Diego, CA, USA). A single colony of A549 Kremen2 knockout cells was picked and expanded, and validated by sequencing and western blot. The CRISPR gRNA of SOCS3 was designed by CRISPick (www.broadinstitute.org) and cloned into the lentiviral vector. The sgSOCS3 sequence was 5′-GGCACGGGCTGCGTGCTCCG-3′. Stably transduced A549 cell populations were selected with 2 μg/ml puromycin for 7 days.

### Knockdown experiment

Small interfering RNAs (siRNAs) were synthesized by GenePharma Co., Ltd. (Shanghai, China) and transfected using Lipofectamine RNAiMax (Invitrogen, Carlsbad, CA, USA) at a siRNA concentration of 20 nM. The siRNA sequences were: Kremen2-targeting siRNA (siKrm2#1) sense sequence (5′-CGGACUUCCCGGACGAGUATT-3′), (siKrm2#2) sense sequence (5′-CGAAUGCUUCCAGGUGAAUTT-3′) and (siKrm2#3) sense sequence (5′- AGGGCUUCCUCUUUCUCCUCUUCCU-3′); EGFR-targeting siRNA (siEGFR#1) sense sequence (5′-GCAGAGGAAUUAUGAUCUUTT-3′) and (siEGFR#2) sense sequence (5′-GGAGAUAAGUGAUGGAGAUTT-3′).

### Lentiviral transduction and plasmid construction

The short hairpin RNA (shRNA) sequence was Kremen2-targeting shRNA (shKrm2): 5′-CCGGCCCGGACTTCCCGGACGAGTACTCGAGTACTCGTCCGGGAAGTCCGGGTTTTT-3′. Lentiviruses containing shRNA or the Kremen2 coding sequence (OE-Krm2) were produced by Shanghai Genechem Co., Ltd. (Shanghai, China). Cells in the logarithmic phase of growth were plated and cultured overnight to reach 30% confluency for lentiviral infection. The cells and viruses were incubated for 8 h, the medium was replaced, and the incubation was continued for 24–48 h. Then, the lentivirus-infected cells were screened with 2 μg/ml puromycin. The cells were seeded into a 6-well plate at 60% confluence for transient expression of Kremen2, and were transfected with the Kremen2 plasmid (Flag-Krm2) using Lipofectamine 2000 (Thermo Fisher Scientific, Waltham, MA, USA) according to the manufacturer’s protocol and collected or treated 24–48 h post-transfection.

### Immunohistochemical (IHC) staining

The paraffin samples used to detect Kremen2 were cut into 4-μm slides and hydrated. Antigen retrieval was performed by heating the slides in 1 × citrate buffer (Abcam, Cambridge, MA, USA) for 10 min. After cooling, the slides were washed with 1 × PBS (pH7.4), and a peroxidase blocking agent (Beijing Zhongshan Golden Bridge Biotechnology Co. Ltd., Beijing, China) was added to the tissue surface and incubated at room temperature for 20 min to block endogenous peroxidase activity. The samples were stained with the Kremen2 antibody (Sigma, St. Louis, MO, USA). The staining was scored as the intensity of positive staining (0—negative, 1—weak, 2—moderate, and 3—strong) multiplied by the stained area (0, < 5%; 1, 5–25%; 2, 26–50%; 3, 51–75%; 4, > 75%). These scores were independently determined by two pathologists. IHC staining was performed to measure Ki67 expression in xenograft tumor nodules on the slides, as described above.

### Colony formation and EdU assays

A total of 500 treated A549 cells were seeded in 6-well plates and 1,500 treated H1703 cells were seeded in 6-well plates for the colony formation assay. Then, the cells were cultured in complete medium for 9 days. The cell colonies were stained with 0.25% crystal violet for 15 min at room temperature. A total of 1.5 × 10^5^ cells were seeded in a 24-well plate with a treated coverglass in advance of the cell growth assay. After the cells adhered, the cells on the slides were removed for EdU staining using the EdU In Vitro Kit (RiboBio, Beijing, China).

### Celigo cell-counting assay

Cells in the logarithmic growth phase were seeded in 96-well plates at 37 °C in a 5% CO_2_ air atmosphere. The Celigo Imaging Cytometer (Nexcelom, Lawrence, MA, USA) was used to measure the number of cells per well for 5 consecutive days.

### Analyses of apoptosis by flow cytometry

The cells pretreated with siRNA were replated in 6-well plates after 8 h of transfection. The cells were harvested after 72 h of transfection and washed once with phosphate-buffered saline. Apoptosis was determined using the FITC Annexin V apoptosis detection kit (BD Pharmingen, San Diego, CA, USA).

### Caspase 3/7 activity assay

Caspase 3//7 activity was detected using Caspase-Glo® 3/7 Assay kit (Promega, USA). Caspase-Glo 3/7 buffer and Caspase-Glo 3/7 substrate was completely dissolved at room temperature and protected from light to form Caspase-Glo Reagent. The cell suspension concentration was adjusted to 1 × 10^4^ cells/well at room temperature. Add 100 µl of cell suspension and 100 µl of Caspase-Glo 3/7 Reagent to each well of a white-walled 96-well plate and mixed gently with a plate shaker at 300–500 rpm for 30 s. Incubate for 30 min to 3 h at room temperature and measure the signal intensity with microplate reader (Tecan, Switzerland).

### Transwell and wound healing assays

The cells for the Transwell assay were seeded into 24-well Transwell chambers at a density of 1 × 10^5^ cells in 200 µl serum-free medium with an 8-μm pore size polycarbonate membrane (Corning, Corning, NY, USA). A 4% paraformaldehyde solution was used to fix the migrated cells, which were stained with 0.25% crystal violet. The cells for the wound-healing migration assay were seeded in 24-well plates and grown until they reached full confluence in RPMI-1640 with 2% FBS. A sterile tip was used to create a “wound” in the cells. Wound closure was measured to assess the migration rate of the cells in the wound field.

### RNA extraction and quantitative real-time polymerase chain reaction (qRT-PCR)

RNA was extracted using TRIzol reagent (Invitrogen) according to the manufacturer’s instructions. Then, total RNA was reverse-transcribed into cDNA using the Prime Script RT Reagent Kit (TaKaRa, Shiga, Japan) and qRT-PCR was performed with the Opticon System (Bio-Rad, Hercules, CA, USA) using SYBR Green Master Mix (ABM, Milton, ONT, Canada). GAPDH was used as a reference for normalization. The primers were synthesized by Sangon Biotech (Shanghai, China) and the primer sequences were:

Kremen2 (F: 5′-GCGCACAACTTCTGCCGTAAC-3′; R: 5′-GTGCCCCTGAGTCCACAAAGC—3′); Snail1 (F: 5′-TGCCCTCAAGATGCACATCCGA-3′; R: 5′- GGGACAGGAGAAGGGCTTCTC-3′); Twist1 (F: 5′-GCCAGGTACATCGACTTCCTCT-3′; R: 5′-TCCATCCTCCAGACCGAGAAGG -3′); ZEB1 (F: 5′-GGCATACACCTACTCAACTACGG-3′; R: 5′-TGGGCGGTGTAGAATCAGAGTC-3′); Vimentin (F: 5′-GCGTGAAATGGAAGAGAAC3′; R: 5′-TGGAAGAGGCAGAGAAATC-3′); N-cadherin (F: 5′-CATCCTGCTTATCCTTGTG-3′; R: 5′-TAGTCCTGGTCTTCTTCTC-3′); MMP2 (F: 5′-AGCGAGTGGATGCCGCCTTTAA-3′; R: 5′- CATTCCAGGCATCTGCGATGAG-3′); MMP9 (F: 5′-GCCACTACTGTGCCTTTGAGTC-3′; R: 5′-CCCTCAGAGAATCGCCAGTACT-3′); EGFR (F: 5′-AACACCCTGGTCTGGAAGTACG-3′; R: 5′-TCGTTGGACAGCCTTCAAGACC-3′).

### Western blot

Total proteins were extracted in RIPA lysis buffer with protease inhibitors and incubated on ice for 30 min. The cell lysate supernatant was supplemented with 4 × loading buffer and boiled for 10 min. Proteins were separated by 8% or 10% sodium dodecyl sulfate–polyacrylamide gel electrophoresis (SDS-PAGE), transferred to nitrocellulose membranes (0.45 µm, Bio-Rad), and nonspecific binding was blocked with 1% bovine serum albumin (BSA) for 1 h at room temperature. The membranes were incubated with specific primary antibodies overnight at 4 °C and with secondary antibodies for 1 h at room temperature. The primary antibodies used in this study include Kremen2 (SAB1305119, Sigma), STAT3 (ab119352, Abcam), p-STAT3 (Y705) (ab76315, Abcam), JAK2 (ab108596, Abcam), p-JAK2 (Y1007 + Y1008) (ab32101, Abcam), LRP6 (#2560, CST), p-LRP6 (Ser1490)(#2568, CST), β-catenin (ab32572, Abcam), EGFR (sc-120, Santa Cruz), p-EGFR (Tyr1068) (#2234, CST), SOCS3 (ab16030 and ab236519, Abcam), c-Myc (ab32072, Abcam), CyclinD1 (ab226977, Abcam), PI3K (#4257, CST), p-PI3K (Y607) (ab182651, Abcam), AKT (#9272, CST), p-AKT (Ser473) (#4060,CST), GAPDH (60,004–1-Ig, Proteintech), Flag (F1804, Sigma), HA (51,064–2-AP, Proteintech).

### Immunoprecipitation assay

Immunoprecipitation (IP) was performed using the A549, H1703, and HEK 293 T cells. The protein lysates were extracted with IP lysis buffer (Thermo) containing a cocktail of phosphatase inhibitors (Roche, Basel, Switzerland) and PMSF (Sigma) for 30 min on ice, followed by centrifugation at 12,000 × g for 15 min. The proteins in the lysates were incubated with protein A/G magnetic beads (Thermo) and the indicated primary antibody or normal IgG overnight at 4 °C. The beads were washed three times in washing buffer. Loading buffer (2 ×) was mixed with the beads and boiled for 10 min. Then, the samples were used for western blot analysis.

### Ni–NTA pulldown

Transiently transfected cells were collected, and the pellets were washed once in ice-cold PBS. The cells were lysed in a buffer containing 150 mM NaCl, 10 mM imidazole, 0.05% Triton-100, and 0.02 M Tris–HCl (pH 8.0), and centrifuged at 10,000 × g for 10 min. The lysates were incubated with Ni–NTA agarose beads (QIAGEN, Hilden, Germany) for 2 h at 4 °C. After washing three times, the beads were boiled in a loading buffer and subjected to SDS-PAGE and western blot.

### Immunofluorescence staining

The cells were seeded onto glass coverslips, washed with PBS, fixed in 4% formaldehyde for 15 min, permeabilized with 0.3% Triton X-100 for 15 min, blocked with 1% BSA for 1 h, and incubated with primary antibodies overnight at 4 °C and with secondary antibodies for 1 h at room temperature. The localization of Kremen2 and SOCS3 was visualized by confocal microscopy.

### Animal experiments

Male athymic nude mice (5 weeks old) were purchased from the Beijing Huafukang Bioscience Co., Ltd. (Beijing, China). A total of 1 × 10^7^ A549 with shCtrl or shKrm2 cells suspended in 100 µl of PBS were subcutaneously injected into the thighs of the nude mice (12 mice per group). And A549 with Ctrl or sgSOCS3 cells were inoculated in the same way (9 mice per group). The sizes of the tumors were measured every 2 days, 7 days after inoculation. Tumor volume = length × width × width/2. When the tumor volume of all groups was 1,000 mm^3^, the tumor-bearing mice were euthanized, and the tumor tissues were collected, weighted, and photographed. The mice for the lung metastasis model with A549 cells (WT), Kremen2 knockout cells (Krm2-KO) and Kremen2 knockout cells reintroduced with Krm2 (Krm2-KO + Krm2) were sacrificed 6 weeks after inoculation in the tail vein and lung tissues were obtained (4 mice per group). Likewise, A549-Ctrl cells and A549-sgSOCS3 cells, A549-OE-Ctrl cells and A549-OE-Kremen2 cells were injected into the mice for the lung metastasis model (7 mice per group), respectively. The number of metastatic lung nodules was counted, and metastatic lung tissues were analyzed by hematoxylin and eosin (H&E) staining. A total of 5 × 10^6^ A549 cells transduced with shCtrl or shKrm2 with GFP in 150 µl of PBS were injected intravenously into the nude mice (4 mice per group) through the tail vein for the lung metastasis model. On day 38, the nude mice were sacrificed and the lung tissues were collected, subjected to fluorescence imaging, and the lung fluorescence signals were counted.

### Database analysis

The lung adenocarcinoma (LUAD) gene expression datasets were analyzed via The Cancer Genome Atlas (TCGA) database. Kremen2 expression in NSCLC was analyzed with Gene Expression Profiling Interactive Analysis (GEPIA) software (http://gepia.cancer-pku.cn/). Kaplan–Meier survival curves were generated using the Kaplan–Meier plotter (http://kmplot.com/analysis/).

### Statistical analysis

All in vitro and in vivo experiments were performed independently at least three times in triplicate. Student’s *t*-test was used to detect differences with GraphPad Prism 8.0 (GraphPad Software Inc., La Jolla, CA, USA) or SPSS software (SPSS Inc., Chicago, IL, USA). The quantitative results are presented as mean ± SEM. A *P* < 0.05 was considered significant (* *P* < 0.05, **P < 0.01, ***P < 0.001, ns: not significantly different).

## Results

### Kremen2 is upregulated in NSCLC and contributes to the poor prognosis

**Fig. 1:**
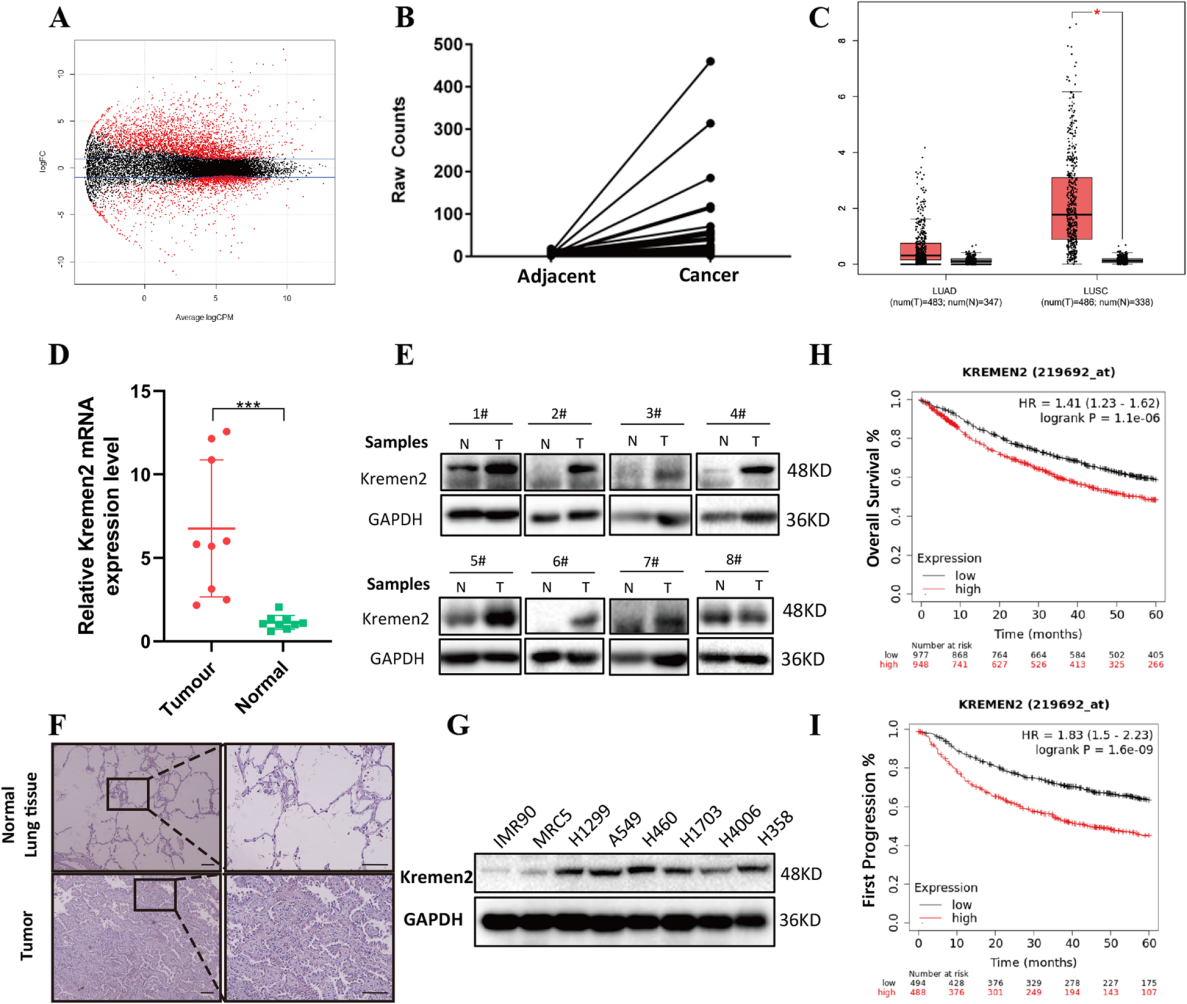
Kremen2 is upregulated in NSCLC and contributes to the poor prognosis. **A** Heatmap of differentially expressed genes filtered in the TCGA database of LUAD patients (red points, *P* < 0.05). **B** The differential expression of Kremen2 in the raw TCGA RNA-seq data (*n* = 57, the y-axis is the raw expression data for each sample). **C** Kremen2 mRNA expression in normal lung tissues, LUSC, and LUAD tissues was analyzed based on the GEPIA profiling datasets (T, tumor tissues; N, normal tissues). **D, E** Kremen2 mRNA (*n* = 9) (**D**) and Kremen2 protein (**E**) expression levels were detected in normal lung tissues and paired NSCLC tissues (*n* = 8). **F** Representative images from IHC staining of Kremen2 in NSCLC (*n* = 12) and matched adjacent tissues (scale bars: 100 μm). **G** Kremen2 protein expression in different human normal lung cell lines and NSCLC cell lines. **H, I** Kaplan–Meier curves of overall survival and first progression survival analysis showing the survival of NSCLC patients with different Kremen2 gene expression levels (HR, hazard ratio)

To identify novel oncogenic genes associated with NSCLC, we searched 504 LUAD samples with pathological information in the TCGA database and screened 57 paired non-cancerous and cancerous samples. The RNA-seq results of these paired patient samples were further analyzed, and differentially expressed genes (DEGs) were screened with a general linear model (*P* < 0.05) (Fig. 1A). The analysis revealed significantly different Kremen2 expression, which has not been studied in lung cancer. Additionally, the Kremen2 expression level was higher in tumor tissues after comparing the raw Kremen2 RNA-seq data for each pair of TCGA samples (Fig. 1B). Furthermore, the expression of Kremen2 in NSCLC was analyzed with GEPIA software (http://gepia.cancer-pku.cn/). The results showed that Kremen2 expression was significantly upregulated in NSCLC tissues, particularly in lung squamous cell carcinoma (LUSC) tissues, compared with adjacent normal lung tissues (*P* < 0.05, Fig. 1C).

To assess Kremen2 expression in NSCLC clinical specimens, we measured Kremen2 mRNA levels in freshly frozen NSCLC specimens and their paired normal tissues. The results showed that the Kremen2 mRNA level was significantly higher in NSCLC tissues than in normal tissues (Fig. 1D). Elevated levels of the Kremen2 protein were detected in most NSCLC specimens by western blot (Fig. 1E). IHC staining further showed that Kremen2 expression was high in tumor tissues (Fig. 1F). In addition, we examined the Kremen2 protein levels in some of the NSCLC cell lines and found that Kremen2 protein expression was generally higher in the NSCLC cells (Fig. 1G).

To elucidate the relevance between Kremen2 overexpression and the survival of NSCLC patients, we analyzed NSCLC cases using the Kaplan–Meier plotter database. The results are shown in Fig. 1H and I, and indicate that NSCLC patients with high Kremen2 expression levels were associated with poorer overall survival (OS), as shown by a lower OS and first progression survival (*P* < 0.05). The chi-square test revealed that upregulated Kremen2 expression was correlated with an advanced primary tumor (T) stage (*P* = 0.041) and pathological grade (*P* = 0.045) (Table 1). Taken together, these data suggest that Kremen2 is overexpressed in NSCLC and that Kremen2 overexpression might be associated with a poor patient prognosis.

**Table 1:** Comparison of clinical features between LUAD patients with low and high Kremen2 levels in TCGA database

Clinical character	Clinical groups	*Kremen2 protein expression*		*P-value*
		**Low(*n* = 248)**	***High(250***^a^***)***	
T stage	T1	90	75	0.041*
	T2	135	132	
	T3	14	31	
	T4	9	9	
Pathological Grade	Stage I	144	128	0.045*
	Stage II	62	57	
	Stage III	32	51	
	Stage IV	10	14	

*P < 0.05

^a^Among them, three samples have no T stage information but have pathological grade information

### Silencing of Kremen2 expression in NSCLC cells inhibits cell proliferation

**Fig. 2:**
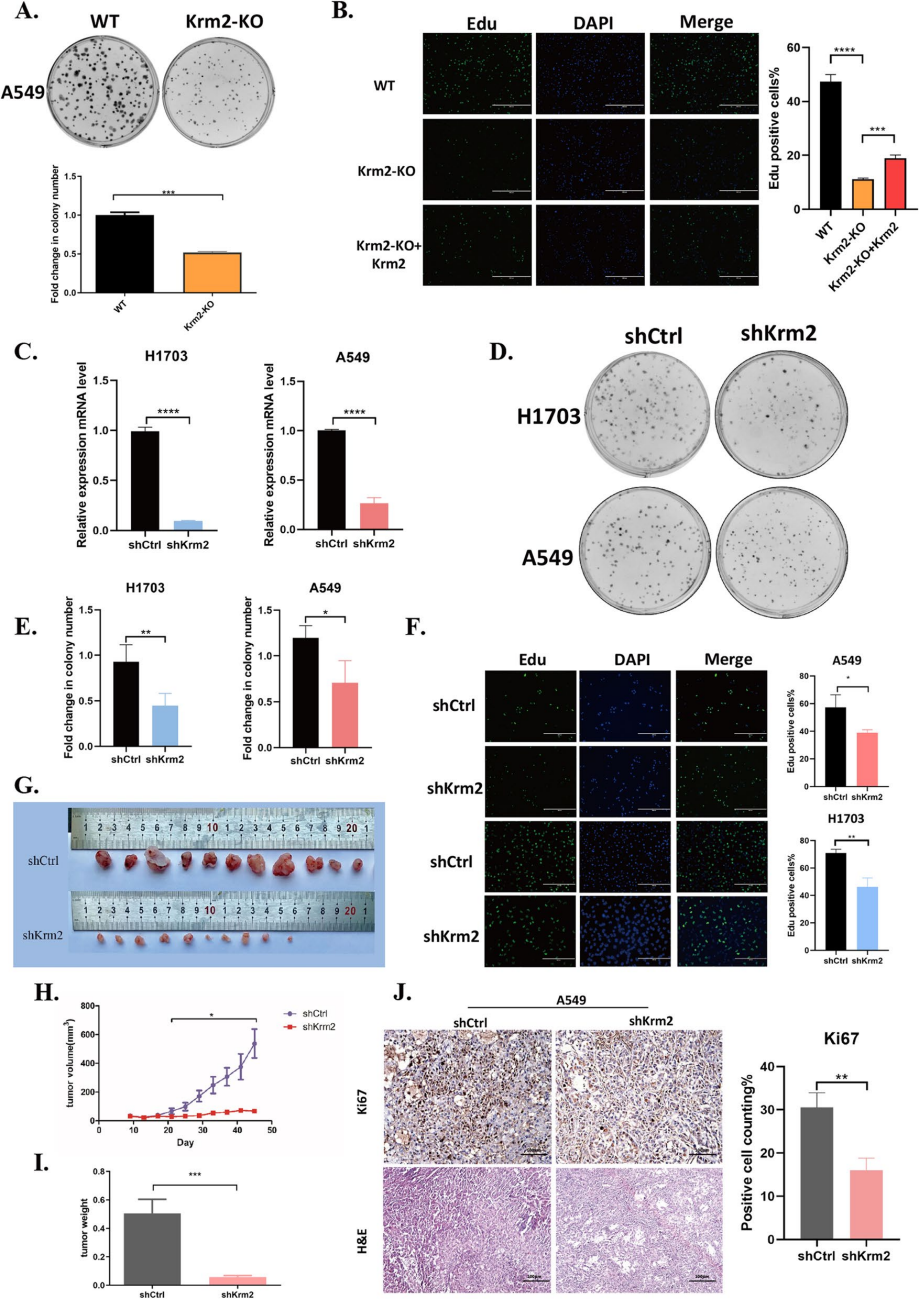
Silencing of endogenous Kremen2 expression in NSCLC cells inhibits cell proliferation. **A** Colony formation assays were conducted using A549-Krm2^KO^ cells. **B** Cell proliferation of A549-Krm2^KO^ and A549-Krm2^KO^ + Flag-Krm2 was detected by the EdU assay. **C** Kremen2 expression was stably silenced using shRNA; Kremen2 mRNA expression levels in H1703 and A549 cells were detected by qPCR. **D, E** The effects of Kremen2 knockdown on colony formation in H1703 and A549 cells. Representative images of the colony formation (**D**) and clone formation rates (**E**). **F** Cell proliferation was measured by the EdU proliferation assay with/without Kremen2 knockdown in H1703 and A549 cells. **G** A subcutaneous xenograft model of nude mice was established using A549 cells with stable Kremen2 knockdown (*n* = 12 per group). **H, I** Tumor volume curve (**H**) and final tumor weight (**I**). **J** IHC analysis of Kremen2 expression on the xenografts (scale bars: 100 μm). The Ki67-positive results (%) are shown in the right panel. Bars represent the mean ± SD, *P < 0.05, **P < 0.01, ***P < 0.001, ****P < 0.0001

To investigate the biological functions of Kremen2 in NSCLC, we utilized the CRISPR/Cas9 approach to generate Kremen2 knockout A549 cells (A549-Krm2^KO^), in which a thymidine residue was inserted leading to a frameshift mutation (Fig. S1A). The lack of the Kremen2 protein in A549 knockout cells was confirmed by western blotting (Fig. S1B). To examine the effects of Kremen2 in the proliferation of NSCLC cells, we performed the colony formation and EdU incorporation assays with A549 and A549-Krm2^KO^ cells. A549-Krm2^KO^ cells exhibited less clonogenic ability than wild-type cells (Fig. 2A) and significantly fewer EdU-positive cells, indicating decreased cell proliferation, while the proportion of EdU-positive cells increased when exogenous Flag-Kremen2 (Flag-Krm2) was overexpressed in A549-Krm2^KO^ cells (Fig. 2B), confirming the effects of Kremen2 on cell proliferation. In addition, we established a stable Kremen2 knockdown NSCLC cell line using lentivirus-mediated shRNA to silence endogenous Kremen2 expression. The efficiency of shRNA transduction was about 90% (Fig. S1C). Kremen2 knockdown was successful as shown by the mRNA (Fig. 2C) and protein levels (Fig. S1D). Similarly, Kremen2 knockdown in A549 and H1703 cells (shKrm2) inhibited colony-formation and cell proliferation abilities (Fig. 2D–F). Furthermore, Kremen2 knockdown of A549 cells suppressed cell growth as assessed by Celigo imaging cytometry (Fig. S1E). We further established the Kremen2 overexpressing A549 cells. The overexpression of Kremen2 was confirmed by western blot (Fig. S2A). Colony-formation assay showed that overexpression of Kremen2 in A549 cells significantly promoted cell proliferation (Fig. S2B). siRNA-mediated silencing of Kremen2 (siKrm2) increased the rate of apoptosis in A549 and H1703 cells (Fig. S1F). In addition, Kremen2 knockdown by shKrm2 increased the activity of Caspase 3/7, indicating the elevated apoptosis level in A549 cells (Fig. S1G). A xenograft model of nude mice was generated by subcutaneously injecting A549 cells with stable Kremen2 knockdown to verify whether these in vitro findings were relevant to NSCLC tumor growth in vivo. Consistent results were obtained from the A549 xenograft model, in which knocked down expression of Kremen2 substantially inhibited tumor size and tumor weight (Fig. 2G–I). IHC staining showed that the percentage of Ki67-positive cells was lower in A549 implanted-mice tissues due to Kremen2 knockdown of A549 cells (Fig. 2J). Taken together, these data demonstrate that Kremen2 plays an important role in NSCLC cell growth.

### High Kremen2 expression promotes NSCLC cell metastasis

**Fig. 3:**
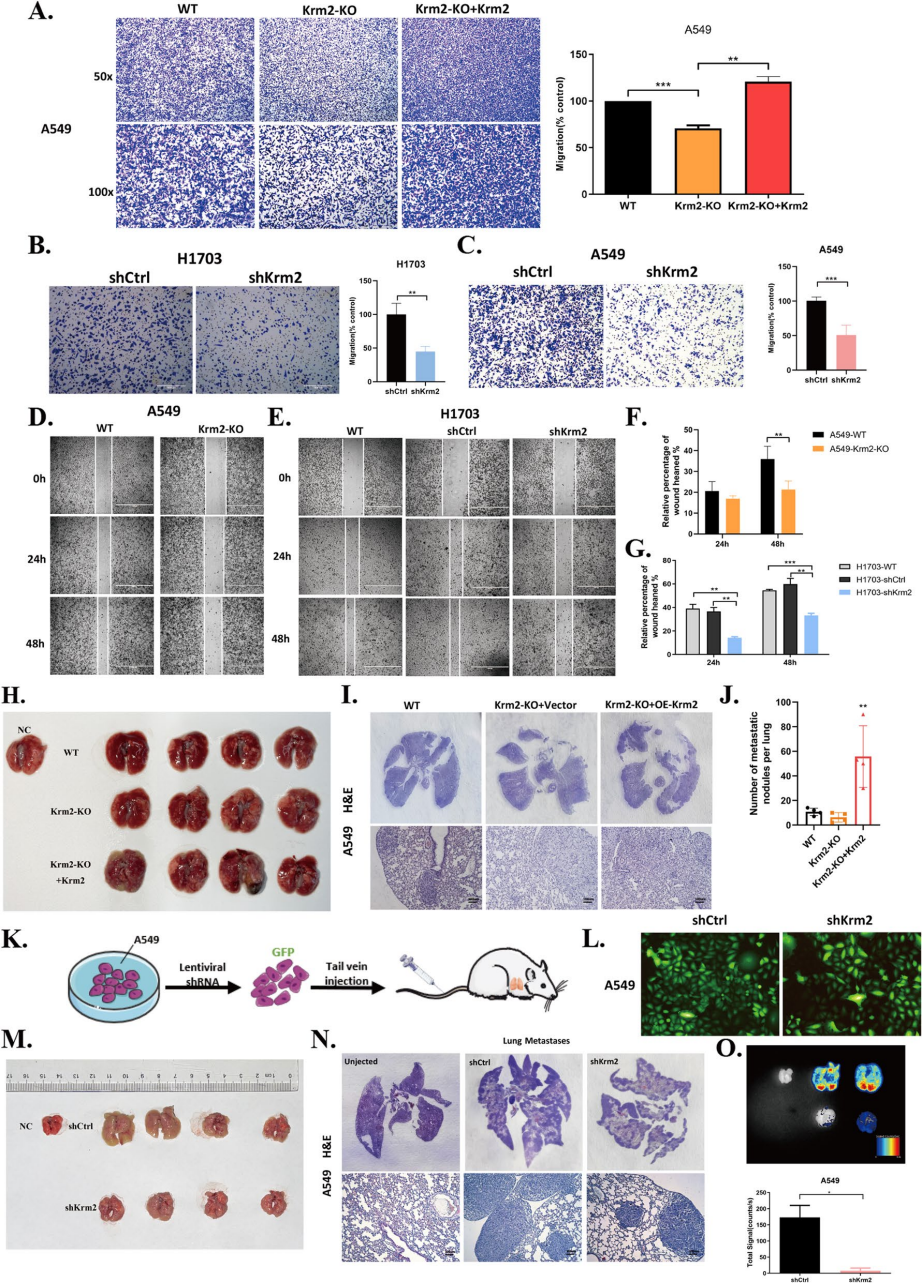
Kremen2 promotes NSCLC cell metastasis in vivo and in vitro. **A** The migration ability of A549-Krm2^KO^ + Flag-Krm2 cells was detected by the Transwell assay. **B** and **C** Transwell migration assays were carried out after the Kremen2-knockdown of H1703 (**B**) and A549 (**C**) cells. Representative images and corresponding statistical analysis of the number of migrating cells. **D–G** Cellular migration and growth were analyzed by the scratch assay in A549 (**D**) and H1703 (**E**) cells. Corresponding statistical analysis of the number of migrating cells (**F, G**). **H, I** Representative images (**H**) and H&E staining (**I**) of the lungs from mice transplanted with A549-Krm2^KO^ cells or A549-Krm2^KO^ + Krm2 cells (*n* = 4) (scale bars: 100 μm).** J** The number of metastatic nodules per lung was measured. **K** Schematic diagram showing construction of the tail vein lung metastasis model using A549 cells with shCtrl-GFP or shKrm2-GFP. **L** Comparison of the GFP fluorescence signal intensity in shCtrl and shKrm2 cells. **M, N** Representative images (**M**) and H&E staining (**N**) of the lungs from mice transplanted with shCtrl-GFP or shKrm2-GFP cells (*n* = 4) (scale bars: 100 μm). **O** Fluorescence luminescence imaging of lung metastases. Fluorescence signal intensity histogram (lower panel). Bars indicate mean ± SD. *P < 0.05, **P < 0.01, ***P < 0.001, ****P < 0.0001

Our clinical data show that Kremen2 expression was positively correlated with LUAD patient pathological grade, suggesting that Kremen2 may be involved in cancer metastasis. To address this issue, we examined the effect of Kremen2 on the metastatic ability of tumor cells in vitro and in vivo. The Transwell assay results showed that deleting Kremen2 attenuated cell invasion in A549 cells, whereas overexpression of Flag-Krm2 in A549-Krm2^KO^ cells enhanced cell invasive ability (Fig. 3A). Similarly, the number of invading H1703 and A549 cells decreased after downregulation of Kremen2 expression by shRNAs (Fig. 3B, C). Moreover, data from the wound healing assay demonstrated that Kremen2 knockdown decreased cell migration (Fig. 3D–G). Conversely, overexpression of Kremen2 promoted cell migration in A549 cells (Fig. S2C). To explore whether Kremen2 inhibited the epithelial-mesenchymal transition (EMT) in NSCLC cells, we analyzed some EMT biomarkers by RT-qPCR after Kremen2 knockdown. A perceived positive correlation was detected between Kremen2 knockdown and low pro-EMT Snail1 and Twist1 levels. However, other EMT biomarkers, such as vimentin, ZEB1, and MMP2, did not change significantly with Kremen2 in A549 cells (Fig. S3). To further understand whether Kremen2 promotes tumor metastasis in vivo, we performed a tail vein metastasis experiment with A549-Krm2^KO^ cells and A549-Krm2^KO^ + OE-Krm2 cells (A549-Krm2^KO^ cells overexpressing Kremen2). The results showed that deleting Kremen2 reduced the number of metastatic lung nodules while overexpressing Kremen2 significantly increased the number of metastatic lung nodules (Fig. 3H–J and Fig. S2D-F). To better observe the formation of metastatic nodules in the lung, we constructed shCtrl and shKrm2 cells co-expressing GFP in A549 cells and observed the fluorescence intensity of the cells (Fig. 3K and L). The two kinds of GFP-expressing cells were injected into the tail veins of nude mice. The H&E staining and fluorescence signal results showed that downregulating Kremen2 in A549 cells decreased the number of pulmonary metastatic nodules compared with the control group (Fig. 3M–O). Taken together, these findings suggest that Kremen2 acts as a facilitator of NSCLC metastasis.

### Kremen2 is involved in multiple cancer-related signaling pathways

**Fig. 4:**
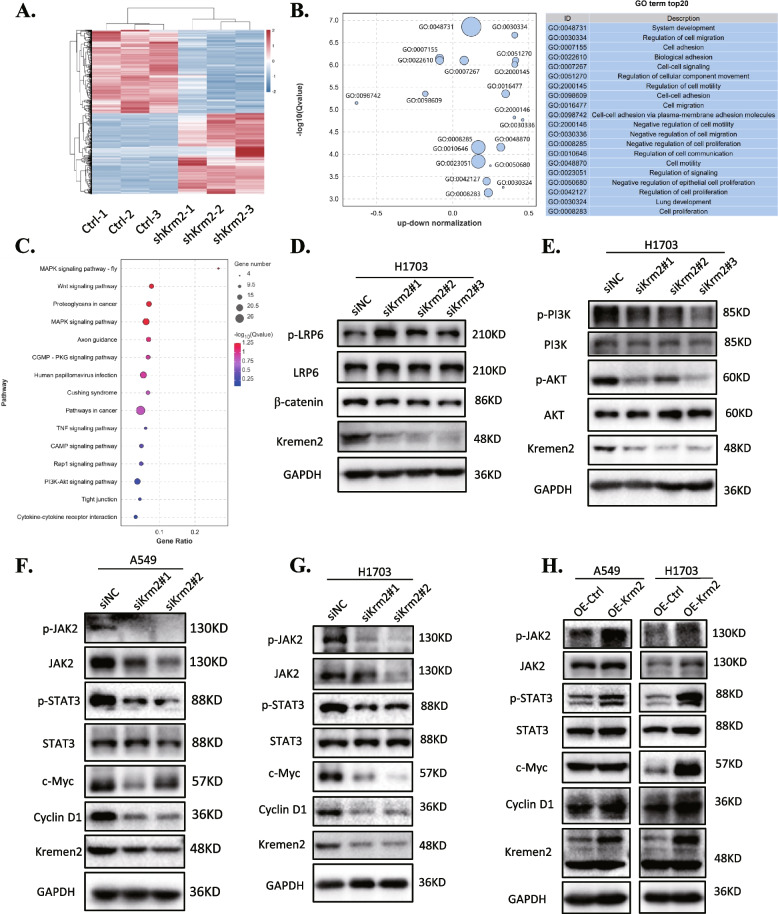
The inhibition of Kremen2 is involved in several cancer-related signaling pathways. **A** Hierarchical clustered heatmap displaying the differentially expressed genes. **B** The KEGG pathway analysis reveals significantly enriched signaling pathways in the differentially expressed genes. **C** GO biological processes show the Z-score normalized expression of the differentially expressed genes in H1703 cells after Kremen2-knockdown according to the RNA-seq data, which were mainly enriched in biological processes, such as cell proliferation and migration. **D, E** Protein levels of Wnt and PI3K/AKT signaling as indicated in the H1703 cell line with Kremen2 knockdown were detected by western blot. **F–H** Effects of Kremen2 level on the expression of proteins associated with cell proliferation and the JAK2-STAT3 signaling pathway in A549 and H1703 cells

To clarify the mechanism of Kremen2 promoting lung cancer, RNA-seq was performed to compare the transcriptome changes between the control and the Kremen2 knockdown (shKrm2) groups of H1703 cells. In total, 273 upregulated genes and 281 downregulated genes were identified in the shKrm2 group based on fold changes in expression (≥ 1 or ≤  − 1) (Fig. S4). Hierarchical clustering analysis was carried out on the upregulated and downregulated genes (Fig. 4A). The Gene Ontology (GO) biological process analysis indicated that the DEGs were closely related to cell proliferation, development, motility, and migration (Fig. 4B). The Kyoto Encyclopedia of Genes and Genomes (KEGG) enrichment analysis demonstrated that these DEGs were enriched in the signaling pathways related to cancer, such as MAPK, Wnt, and PI3K-AKT signaling (Fig. 4C). As previous Kremen2 studies have mostly focused on the Wnt signaling pathway [5, 28], we first detected changes in the expression of key proteins in the Wnt signaling pathway. The western blot results showed that Kremen2 knockdown did not significantly affect β-catenin protein levels, but increased the total protein and phosphorylation levels of LRP6 in H1703 cells (Fig. 4D), possibly due to reduced endocytic degradation of LRP6 [29]. Furthermore, phosphorylation of PI3K (Y607) and AKT (Ser473) was significantly downregulated after Kremen2 knockdown in H1703 cells (Fig. 4E), which was consistent with the results from a study on Kremen2 in gastric cancer [10]. We also observed the reduced phosphorylation of PI3K (Y607) and AKT (Ser473) in Kremen2 knockdown A549 cells (Fig. S5), suggesting that Kremen2 may regulate the PI3K-AKT signaling pathway.

As activation of the STAT3 pathway has been reported in a significant proportion of NSCLC cases, we also tested whether Kremen2 regulates the STAT3 signaling pathway. The western blot results showed that the phosphorylated JAK2 (Y1007 and Y1008) and STAT3 (Y705) levels were downregulated, while total JAK2 and STAT3 were unchanged. At the same time, the downstream factors in the STAT3 pathway responsible for regulating cell proliferation, such as c-Myc and Cyclin D1, also decreased significantly at the protein level (Fig. 4F and G). In contrast, overexpression of Kremen2 enhanced p-JAK2, p-STAT3, c-Myc, and Cyclin D1 protein levels (Fig. 4H), suggesting that Kremen2 affects the JAK2-STAT3 signaling pathway.

### Kremen2 promotes NSCLC cell proliferation by stabilizing EGFR

**Fig. 5:**
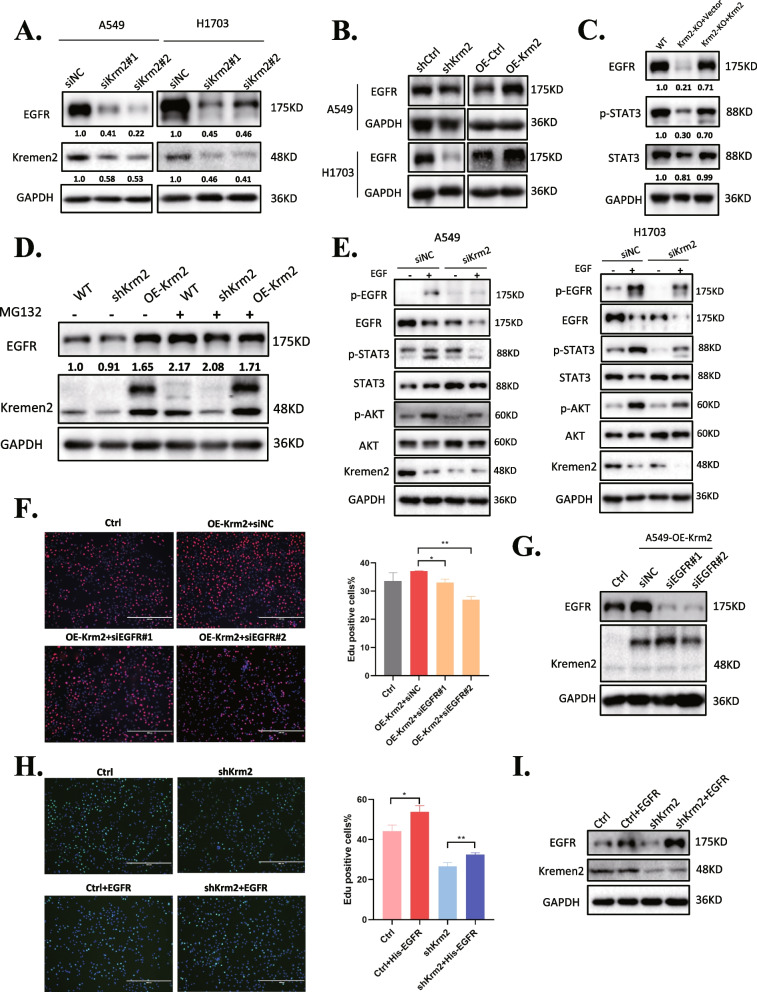
Kremen2 promotes NSCLC cell proliferation by promoting the stability of EGFR. **A** EGFR levels in Kremen2 knockdown cells treated with siRNA. **B** EGFR levels in Kremen2 over-expressing and Kremen2 knockdown of A549 and H1703 cells. **C** EGFR and phosphorylated STAT3 (Y705) protein levels in A549-Krm2^KO^ and A549-Krm2^KO^ + Flag-Krm2 cells. **D** A549 cells stably expressing Kremen2 shRNA or over-expressing Kremen2 (OE-Krm2) were treated with MG132 or not. The cells were lysed, and western blotting was performed with the indicated antibodies. **E** A549 and H1703 cells transfected with siKrm2 small-interfering RNA were stimulated with or without EGF (50 ng/mL) for 15 min. **F, G** Cell proliferation was measured by the EdU proliferation assay (**F**) and the EGFR expression levels were measured in Kremen2 over-expressing A549 cells treated with siNC or siEGFR (**G**). **H, I** Cell proliferation was measured by the EdU proliferation assay (**H**) and EGFR levels were measured in cells transfected with or without the His-EGFR plasmid (**I**)

Considering EGFR is a common upstream activator for the JAK2-STAT3 and PI3K-AKT signaling pathways and plays an important role in the progression of NSCLC [30, 31], we inputted the DEGs screened from shKrm2 H1703 cells into FunRich software (version 3) for functional analysis. As results (Fig. S6) shown, most of the genes were clustered in EGFR-related pathways. Then, we examined whether Kremen2 affected the EGFR protein level. The western blot results indicated that siRNA-mediated silencing of Kremen2 downregulated the EGFR protein levels in A549 and H1703 cells (Fig. 5A). The EGFR protein level also decreased in Kremen2 stable knockdown cells, while overexpression of Kremen2 increased the EGFR protein level (Fig. 5B). We obtained similar results in A549-Krm2^KO^ cells. Reintroducing Flag-tagged Kremen2 into A549-Krm2^KO^ cells restored the EGFR protein levels (Fig. 5C). As Kremen2 affects the EGFR protein level but not the EGFR mRNA level (Fig. S7), we speculated that Kremen2 might affect the degradation of EGFR protein. As shown in Fig. 5D, the decrease in the EGFR protein level in Kremen2-depleted cells was reversed by the proteasome inhibitor MG132, suggesting that Kremen2 may regulate EGFR protein levels via proteasomal degradation. We further examined whether Kremen2 knockdown affected EGFR phosphorylation following stimulation with EGF. The results showed that silencing Kremen2 reduced EGF-induced phosphorylation of EGFR, STAT3, and AKT in A549 and H1703 cells (Fig. 5E). To evaluate whether Kremen2 promotes cell proliferation by regulating EGFR, we depleted endogenous EGFR in Kremen2-overexpressing A549 cells and observed that inhibiting endogenous EGFR expression attenuated Kremen2-induced cell proliferation (Fig. 5F). In contrast, overexpressing EGFR in Kremen2-knockdown A549 cells showed that exogenous EGFR expression promoted the proliferation of Kremen2 knockdown cells (Fig. 5H). EGFR expression was detected in Fig. 5G and I. Collectively, these data suggest that a high expression level of Kremen2 may promote tumor progression by maintaining the EGFR stability in NSCLC.

### Interaction between Kremen2 and SOCS3 inhibits EGFR ubiquitination and degradation

**Fig. 6:**
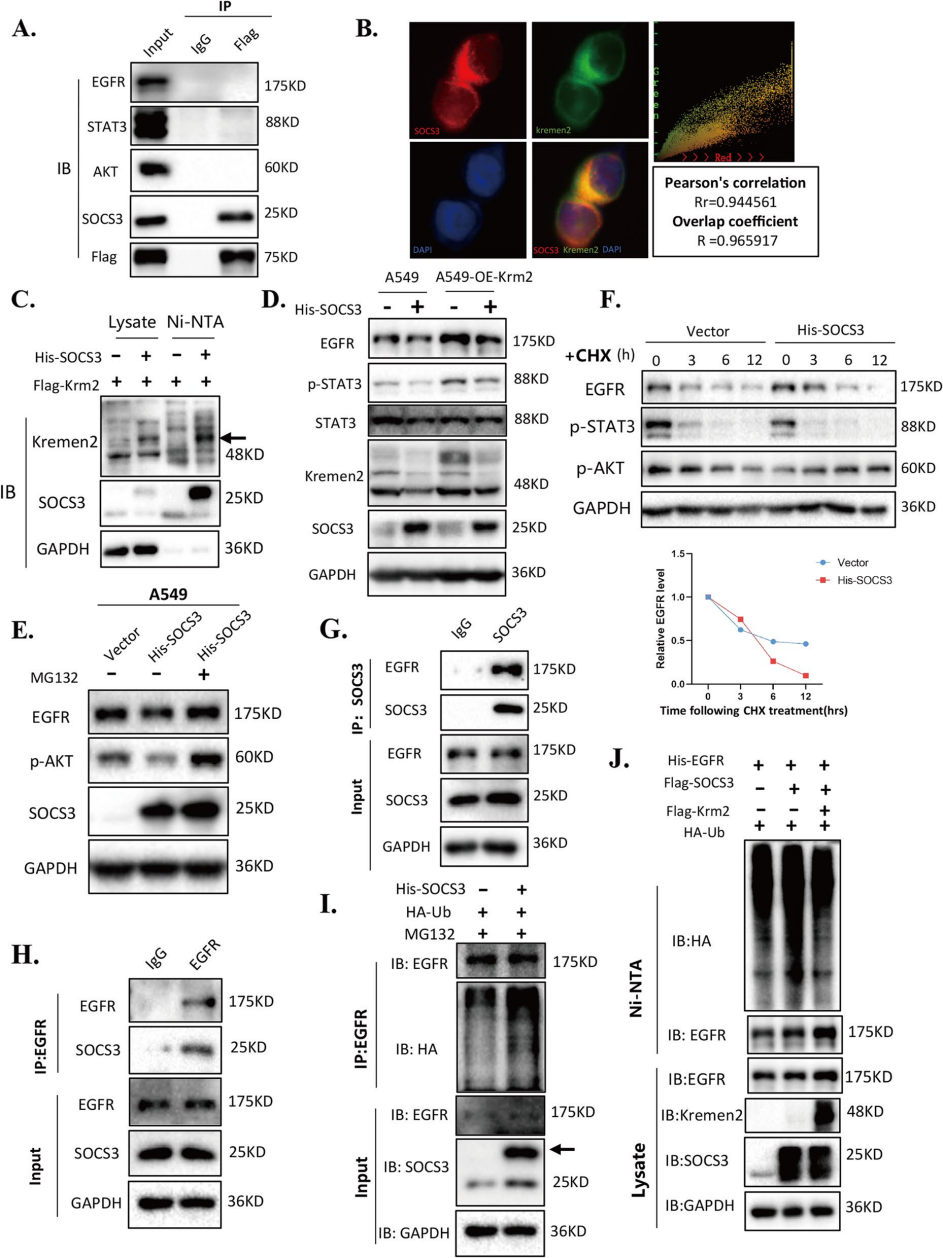
The interaction between Kremen2 and SOCS3 inhibits EGFR ubiquitination and degradation. **A** HEK293T cells were transfected with the Flag-Krm2 plasmid. IB analysis of exogenous Kremen2, EGFR, AKT, STAT3, and SOCS3, as assessed after co-IP with IgG or anti-Flag. **B** Co-localization of red (SOCS3) and green (Kremen2) was analyzed by Image-Pro Plus 6.0. **C** HEK293T cells were transfected with His-SOCS3 and Flag–Krm2. Ni–NTA beads were used to pull down His-tagged SOCS3, and immunoblotted with the indicated antibodies. **D** EGFR expression levels were measured in A549 cells and Kremen2 over-expressing A549 cells transfected with the vector or His-SOCS3 plasmid. **E** A549 cells expressing the vector or His-SOCS3 were treated with MG132 or not. **F** A549 cells stably expressing the vector or His-SOCS3 were treated with CHX (0.1 mg/ml) and collected at the indicated time points. The intensity of EGFR bands in western blot was measured with ImageJ software and normalized to the corresponding GAPDH and plotted. **G, H** IB analysis of the endogenous EGFR and SOCS3 in H1703 cells, as assessed after co-IP with anti–EGFR or anti-SOCS3. **I** A549 cells transfected with the indicated plasmids were treated with 20 μM MG132 for 8 h before co-IP. **J** HEK293T cells were transfected with indicated plasmids and treated with 20 μM MG132 for 8 h. Ni–NTA beads were used to pull down His-tagged EGFR, and the polyubiquitylated EGFR protein was examined

To understand how Kremen2 regulates EGFR, we performed co-IP to pulldown Kremen2-interacting proteins, which are involved in the EGFR signaling pathway. Surprisingly, Kremen2 did not interact with EGFR, AKT, or STAT3 proteins, but was strongly associated with SOCS3, a negative regulator of the STAT3 signaling pathway (Fig. 6A). Furthermore, colocalization of endogenous Kremen2 and SOCS3 was observed in A549 cells (Fig. 6B) and H1703 cells (Fig. S8). Purification of exogenous SOCS3 under denaturing conditions followed by immunoblot analysis of Kremen2 confirmed the interaction between Kremen2 and SOCS3 (Fig. 6C). Next, to explore the role of SOCS3 in the relationship between Kremen2 and EGFR, we overexpressed SOCS3 in A549 and A549-OE-Krm2 cells, respectively. As shown in Fig. 6D, overexpressing exogenous SOCS3 decreased the EGFR protein level and the phosphorylation level of STAT3 in A549 cells, while stably overexpressing Kremen2 partially reversed this phenomenon (Fig. 6D). To further demonstrate the function of SOCS3, we generated SOCS3 knockout A549 cells. Western blot results showed that depletion of SOCS3 remarkably increased the phosphorylation level of STAT3 (Fig. S9A and B) and subsequent knockdown of Kremen2 partially reversed this phenomenon (Fig. S9B). Wound healing and Transwell assays showed that knockout of SOCS3 (sgSOCS3) promoted the migration ability of A549 cells (Fig. S9C and D). EdU and colony formation assays demonstrated that deletion of SOCS3 also promoted the proliferation of A549 cells (Fig. S9E and F). Nevertheless, the overexpression of Kremen2 alleviated the pro-proliferative and metastatic ability of sgSOCS3 cells (Fig. S9D and F). The knockout of SOCS3 also promoted the tumor growth and metastasis in vivo, as evidenced by the increased tumor size and more metastatic lung nodules in animal experiments (Fig. S10).

In addition, the decrease in EGFR level in SOCS3-overexpressed cells was reversed by treatment with the proteasome inhibitor MG132, suggesting that SOCS3 regulates the EGFR level via proteasomal degradation (Fig. 6E). To further investigate how SOCS3 regulates EGFR, we treated cells with cycloheximide (CHX) and examined the half-life of EGFR. The results showed that overexpressing SOCS3 shortened the half-life of the EGFR protein (Fig. 6F). Next, we performed reciprocal co-IP of the endogenous proteins. As expected, endogenous SOCS3 interacted with EGFR (Fig. 6G), and endogenous EGFR bound to SOCS3 (Fig. 6H). These results demonstrate that SOCS3 can bind to EGFR and promote the degradation of EGFR in cells.

SOCS3 has been reported to promote ubiquitination and degradation of TBK1 and integrin β1 [25, 32]; thus, we next investigated the effect of SOCS3 on EGFR ubiquitination. As results, overexpressing SOCS3 in A549 cells increased polyubiquitination of EGFR compared to the control group (Fig. 6I). To further investigate whether Kremen2 affected SOCS3-mediated ubiquitination of EGFR, in vitro ubiquitination assays were carried out. Flag-SOCS3 and His-EGFR were co-expressed in HEK293T cells, and His-EGFR was purified under denaturing conditions, followed by immunoblot analysis of EGFR to demonstrate that exogenous EGFR ubiquitination in SOCS3 overexpressing cells increased, while Kremen2 overexpression resulted in a decrease in polyubiquitination of EGFR by SOCS3 (Fig. 6J).

Taken together, these data suggest that Kremen2 interacts with SOCS3 and stabilizes the EGFR protein by preventing SOCS3-mediated EGFR ubiquitination and degradation; thereby, enhancing the activation of EGFR signaling and promoting cell proliferation and metastasis of NSCLC.

## Discussion

**Fig. 7:**
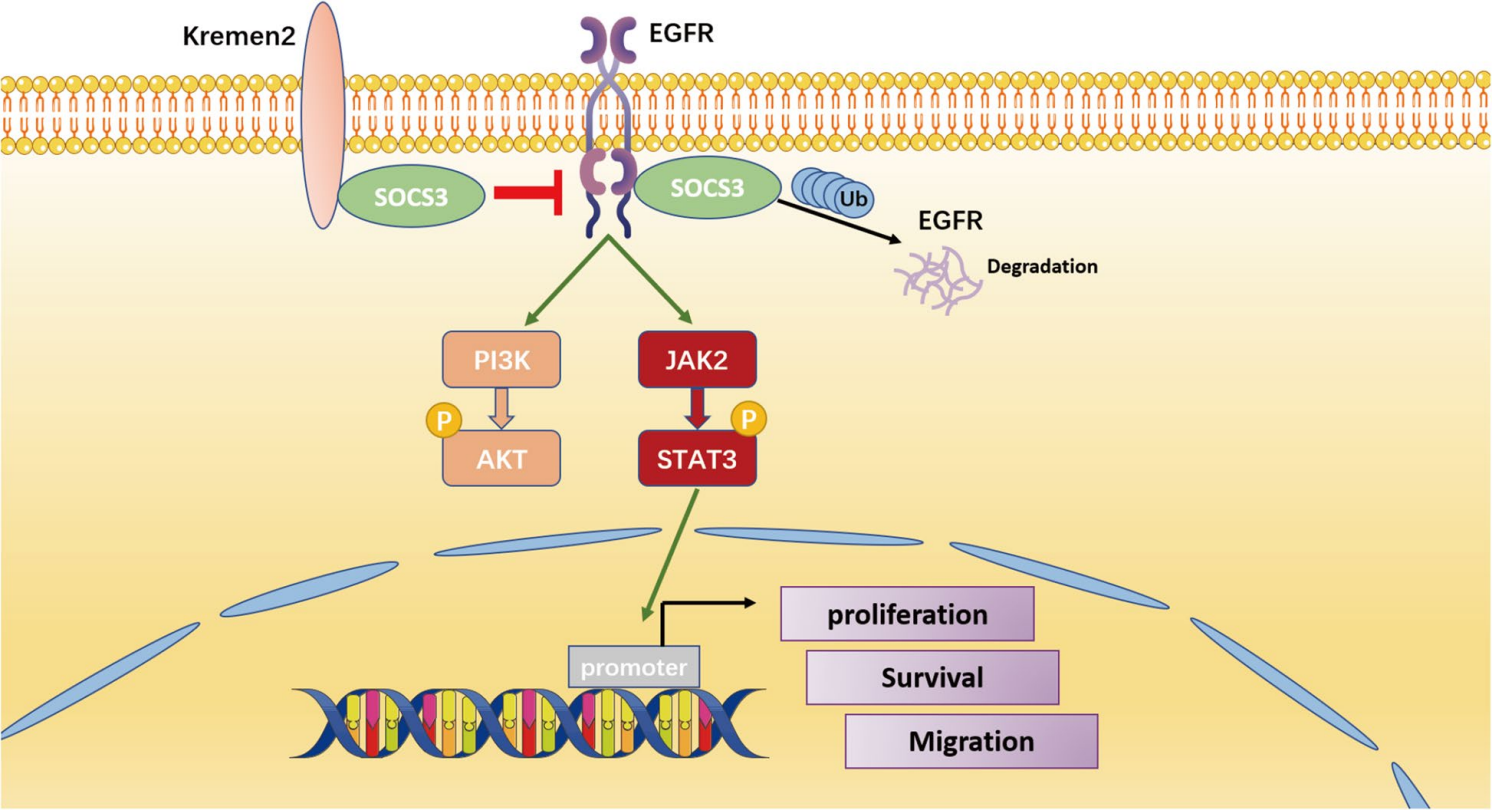
Schematic illustration of the functional roles of Kremen2 in promoting activation of the EGFR signaling pathways by increasing the stability of the EGFR protein and promoting the malignant progression of NSCLC

Although previous studies have described the functions of Kremen2 as a negative regulator of the Wnt signaling pathway in embryonic development and bone formation [28, 33], increasing evidence suggests an oncogenic role of Kremen2 [7, 8, 10]. Here, we comprehensively investigated the role of Kremen2 in NSCLC and found that Kremen2 promoted NSCLC cell proliferation and metastasis in vitro and in vivo. We also studied the molecular mechanism and showed that Kremen2 bound to SOCS3 to inhibit SOCS3-mediated EGFR degradation and promote activation of the EGFR signaling pathway, which exacerbated the malignant progression of NSCLC (Fig. 7).

In the canonical Wnt/β-catenin pathway, the Wnt ligand binds to the transmembrane receptors Frizzled (Fz) and low-density lipoprotein receptor-related protein 5/6 (Lrp5/6), initiating a process that leads to stabilization and activation of β-catenin. DKK1 inhibits the formation of the Wnt-Fz-Lrp5/6 functional complex by binding to Lrp5/6, thereby inhibiting the Wnt/β-catenin signaling pathway [34]. Kremen2, as a high-affinity receptor for DKK1, strongly cooperates with DKK1 to inhibit Wnt/β-catenin signaling. However, in the absence of DKK1, Kremen2 enhances Wnt signaling by maintaining Lrp5/6 on the plasma membrane [35], suggesting DKK1-independent roles for Kremen2. Furthermore, Kremen2 is highly expressed in a variety of tumors, particularly in squamous lung cancer [9], but the expression of Kremen1 is absent in 30 different human tumor cell lines, especially in the human lung cancer cell lines (Lu99, EBC1, SBC2, and SBC5) [36]. It is possible that Kremen2, as a suppressor of Kremen1, inhibits Kremen1-induced cell death and Kremen2 may promote the survival of tumor cells in a ligand-deficient environment [9]. Therefore, Kremen2 has two sides and behaves differently in different environments. In this study, Kremen2 was highly expressed in LUAD according to the TCGA clinical data analysis (Fig. 1B), while the bioinformatics analysis from the GEPIA database further confirmed the high expression of Kremen2 in NSCLC (Fig. 1C). The clinical significance of Kremen2 as an independent prognostic indicator of OS in NSCLC patients was confirmed by combining the correlation between 5-year survival and clinical grade in NSCLC patients in a comprehensive analysis (Fig. 1H and Table 1), indicating that upregulation of Kremen2 expression is related to a poorer prognosis in NSCLC patients. In addition, we consistently detected the expression of Kremen2 in clinical tissue samples (Fig. 1D–F). High expression of Kremen2 enhanced tumor cell growth (Fig. 2) and metastasis (Fig. 3) in NSCLC in vitro and in vivo.

Previous studies have shown that Kremen2 regulates Wnt signaling. Our results revealed no significant changes in the β-catenin protein in NSCLC cells after Kremen2 knockdown (Fig. 4D and Fig. S5). There are two explanations for this discrepancy. First, previous studies on Kremen2 and the Wnt pathway were based on normal cells, and few studies have been performed on cancer cells, especially NSCLC. Second, our experiments only detected changes in the downstream β-catenin protein in the canonical Wnt pathway and did not comprehensively determine whether Kremen2 regulates the Wnt signaling pathway. In our experiments, knockdown of Kremen2 resulted in increased levels of p-LRP6 and LPR6 protein in H1703 cells (Fig. 4D) while the levels of p-LRP6 and LPR6 did not change obviously in A549 cells (Fig. S5), implying Kremen2 might play different roles in different lung cancer cells. In addition to other studies that observed hyperactivation of the PI3K/AKT pathway due to Kremen2 overexpression [10], our transcriptome sequencing results suggested that Kremen2 may also be involved in multiple oncogenic signaling pathways in NSCLC. Kremen2 activates the PI3K/AKT pathway in gastric cancer, and the knockdown of Kremen2 decreases total PI3K and AKT proteins [10], which, in turn, prevents AKT-mediated p53 degradation [37] but promotes apoptosis. That study suggested that Kremen2 might be involved in tumor development by affecting the AKT transcriptional level. However, our results show that the knockdown of Kremen2 in NSCLC did not affect the PI3K and AKT protein levels, but only decreased the PI3K and AKT phosphorylation levels (Fig. 4E and Fig. S5). Thus, this finding indicates that Kremen2 in NSCLC might affect the upstream factors of PI3K and AKT indirectly leading to activation of the PI3K/AKT signaling pathway. Moreover, our results reveal for the first time that Kremen2 promoted activation of the JAK/STAT3 pathway by promoting phosphorylation of JAK2 and STAT3 (Fig. 4F–H). The JAK/STAT3 pathway is aberrantly over-activated in many types of cancer and this over-activation is often associated with a poor clinical prognosis [38]. The phosphorylation of AKT and STAT3 induces the transcription of encoded cell proliferation regulators, such as Cyclin D1 and c-Myc [39, 40]. Consistently, Kremen2 positively regulated the protein expression of Cyclin D1 and c-Myc (Fig. 4F–H).

The EGFR is an RTK that links extracellular signaling to the control of cell survival, proliferation, and differentiation [41]. An EGFR gene mutation is most common in NSCLC, and the frequency of EGFR mutations is as high as 60% in Asian non-smoking NSCLC patients [42]. Previous studies have reported that tumor cells promote cell self-survival through the EGFR-STAT3 and EGFR-AKT pathways [30, 31, 43, 44]. Based on the result that Kremen2 only regulated changes in the phosphorylation AKT and STAT3 levels, we hypothesized that Kremen2 may affect the activation of the AKT and STAT3 signaling pathways by regulating upstream EGFR. In the present study, we examined the possible relationship between Kremen2 and EGFR in the context of NSCLC, and the results showed that Kremen2 affected the EGFR protein level but not its mRNA level (Fig. 5A–C and Fig. S7). Therefore, we speculate that Kremen2 affected the degradation of EGFR. The protein degradation pathway mainly includes proteasomal degradation and autophagic lysosomal degradation. In our study, the proteasome inhibitor MG132 reversed Kremen2 knockdown-induced EGFR degradation, suggesting that Kremen2 stabilizes the EGFR protein (Fig. 5D). Furthermore, Kremen2 was necessary for EGF-induced EGFR, AKT, and STAT3 activation, which is critical for the progression of NSCLC (Fig. 5E). Currently, studies on the ubiquitinated degradation of EGFR are mainly based on inhibiting EGF-induced EGFR degradation by interfering with EGFR binding to c-CBL, which is an E3-ubiquitin ligase that tightly regulates ubiquitination of EGFR [45, 46]. It has been demonstrated that NSCLC-associated EGFR mutants appear to have impaired interactions with c-CBL, causing defective ubiquitination and degradation of EGFR, resulting in prolonged EGFR signaling [47, 48]. However, whether blocking the ubiquitin degradation pathway of EGFR by Kremen2 is related to c-CBL is a question for future studies.

The SOCS family of proteins are negative regulators of cytokine receptor signaling and consist of eight structurally similar proteins, such as SOCS1–7, and the cytokine-induced SH2-containing proteins. The structure of this family of proteins is characterized by an SH2 structural domain and a SOCS box [49]. SOCS proteins have been shown an important role in RTK signaling. These proteins recruit E3 ubiquitin through their SOCS boxes, thereby limiting receptor stability by inducing ubiquitination [18]. Previous studies have reported that SOCS4 and SOCS5 induce ubiquitination of EGFR followed by degradation [50–52]. In addition, SOCS3 binds to the EGFR cytoplasmic structural domain [20]. In our study, we revealed that SOCS3 interacted with EGFR (Fig. 6G–H) and destabilized EGFR through the proteasomal degradation pathway (Fig. 6E–F). Overexpression of SOCS3 promoted the level of EGFR ubiquitination (Fig. 6I). The ubiquitination function of SOCS3 has been demonstrated in other studies [30, 31, 53], SOCS3-mediated ubiquitination of EGFR has not been reported. Although we do not have a clear understanding of the high-affinity site for SOCS3 binding to EGFR, studies of SOCS4/5 have observed a mechanism by which SOCS4/5 inhibits EGFR by binding to the Y1092 site on EGFR [50]. Therefore, we can further explore the significance of this Y1092 site for SOCS3 to promote ubiquitinated degradation of EGFR based on that study. Importantly, we revealed a strong interaction between Kremen2 and SOCS3 through co-IP, immunofluorescence staining, and Ni–NTA pulldown assays (Fig. 6A–C). Furthermore, the Ni–NTA pulldown results demonstrated that overexpressing Kremen2 inhibited the ubiquitination of EGFR by SOCS3 (Fig. 6J). However, the detailed mechanism by which Kremen2 regulates the degradation of the EGFR by SOCS3 remains to be further explored.

## Conclusion

In conclusion, we demonstrated a novel role for Kremen2 in NSCLC tumorigenesis. Mechanistically, Kremen2 may exert its oncogenic effect by interacting with SOCS3 and blocking the ubiquitin-dependent EGFR degradation, thereby sustaining the EGFR-mediated cancer signaling pathways and promoting cell proliferation and metastasis of NSCLC. Therefore, Kremen2 may serve as a new therapeutic target in NSCLC.

## Supplementary Information

**Additional file 1.**

## Data Availability

All analyzed and generated data are presented in the published article and supplementary information. The datasets used and/or analyzed during the current study are available from the corresponding author on reasonable request.

## Abbreviations

Kremen: Kringle containing transmembrane protein
NSCLC: Non-small cell lung cancer
EGFR: Epidermal growth factor receptor
RTK: Receptor tyrosine kinase
SOCS: The suppressor of cytokine signaling
siRNA: Small interfering RNA
shRNA: Short hairpin RNA
qRT-PCR: Quantitative real-time polymerase chain reaction
IP: Immunoprecipitation
TCGA: The Cancer Genome Atlas
GEPIA: Gene Expression Profiling Interactive Analysis
LUAD: Lung adenocarcinoma
LUSC: Lung squamous cell carcinoma
OS: Overall survival
EMT: Epithelial-mesenchymal transition
DEG: Differentially expressed gene
KEGG: Kyoto Encyclopedia of Genes and Genomes
CHX: Cycloheximide
DKK: Dickkopf homolog
Lrp5/6: Low-density lipoprotein receptor-related protein 5/6

## References

[1] Bray F, Ferlay J, Soerjomataram I, Siegel RL, Torre LA, Jemal A (2018). Global cancer statistics 2018: GLOBOCAN estimates of incidence and mortality worldwide for 36 cancers in 185 countries. Cancer J Clin.

[2] Gould MK (2014). Clinical practice. Lung-cancer screening with low-dose computed tomography. N Engl J Med.

[3] Wu C, Li M, Meng H, Liu Y, Niu W, Zhou Y (2019). Analysis of status and countermeasures of cancer incidence and mortality in China. Science China Life sciences.

[4] Hirsch FR, Scagliotti GV, Mulshine JL, Kwon R, Curran WJ, Wu YL (2017). Lung cancer: current therapies and new targeted treatments. Lancet.

[5] Ellwanger K, Saito H, Clément-Lacroix P, Maltry N, Niedermeyer J, Lee WK (2008). Targeted disruption of the Wnt regulator Kremen induces limb defects and high bone density. Mol Cell Biol.

[6] Schulze J, Seitz S, Saito H (2010). Negative regulation of bone formation by the transmembrane Wnt antagonist Kremen-2. PLoS ONE.

[7] Dun X, Jiang H, Zou J, Shi J, Zhou L, Zhu R (2010). Differential expression of DKK-1 binding receptors on stromal cells and myeloma cells results in their distinct response to secreted DKK-1 in myeloma. Mol Cancer.

[8] Maehata T, Taniguchi H, Yamamoto H, Nosho K, Adachi Y, Miyamoto N (2008). Transcriptional silencing of Dickkopf gene family by CpG island hypermethylation in human gastrointestinal cancer. World J Gastroenterol.

[9] Sumia I, Pierani A, Causeret F (2019). Kremen1-induced cell death is regulated by homoand heterodimerization. Cell death discovery.

[10] Chen B, Wang SQ, Huang J, Xu W, Lv H, Nie C (2021). Knockdown of Kremen2 Inhibits Tumor Growth and Migration in Gastric Cancer. Front Oncol.

[11] Concu R, Cordeiro M (2018). Looking for new inhibitors for the epidermal growth factor receptor. Curr Top Med Chem.

[12] Abella JV, Park M (2009). Breakdown of endocytosis in the oncogenic activation of receptor tyrosine kinases. Am J Physiol Endocrinol Metab.

[13] Yu JJ, Zhou DD, Yang XX, Cui B, Tan FW, Wang J (2020). TRIB3-EGFR interaction promotes lung cancer progression and defines a therapeutic target. Nat Commun.

[14] Sundaram K, Senn J, Reddy SV (2013). SOCS-1/3 participation in FGF-2 signaling to modulate RANK ligand expression in paget's disease of bone. J Cell Biochem.

[15] Maity TK, Venugopalan A, Linnoila I, Cultraro CM, Giannakou A, Nemati R (2015). Loss of MIG6 Accelerates Initiation and Progression of Mutant Epidermal Growth Factor Receptor-Driven Lung Adenocarcinoma. Cancer Discov.

[16] Casaletto JB, McClatchey AI (2012). Spatial regulation of receptor tyrosine kinases in development and cancer. Nat Rev Cancer.

[17] Jiang R, Tang J, Chen Y, Deng L, Ji J, Xie Y (2017). The long noncoding RNA lnc-EGFR stimulates T-regulatory cells differentiation thus promoting hepatocellular carcinoma immune evasion. Nat Commun.

[18] Kazi JU, Kabir NN, Flores-Morales A, Rönnstrand L (2014). SOCS proteins in regulation of receptor tyrosine kinase signaling. Cell Mol Life Sci.

[19] Gao Y, Zhao H, Wang P, Wang J, Zou L (2018). The roles of SOCS3 and STAT3 in bacterial infection and inflammatory diseases. Scand J Immunol.

[20] Xia L, Wang L, Chung AS, Ivanov SS, Ling MY, Dragoi AM (2002). Identification of both positive and negative domains within the epidermal growth factor receptor COOH-terminal region for signal transducer and activator of transcription (STAT) activation. J Biol Chem.

[21] Rui L, Yuan M, Frantz D, Shoelson S, White MF (2002). SOCS-1 and SOCS-3 block insulin signaling by ubiqui tin-mediated degradation of IRS1 and IRS2. J Biol Chem.

[22] Sasi W, Jiang WG, Sharma A, Mokbel K (2010). Higher expression levels of SOCS 1,3,4,7 are associated with earlier tumour stage and better clinical outcome in human breast cancer. BMC Cancer.

[23] Niwa Y, Kanda H, Shikauchi Y, Saiura A, Matsubara K, Kitagawa T (2005). Methylation silencing of SOCS-3 promotes cell growth and migration by enhancing JAK/STAT and FAK signalings in human hepatocellular carcinoma. Oncogene.

[24] Zhang L, Li J, Li L, Zhang J, Wang X, Yang C (2014). IL-23 selectively promotes the metastasis of colorectal carcinoma cells with impaired Socs3 expression via the STAT5 pathway. Carcinogenesis.

[25] Zhao G, Gong L, Su D, Jin Y, Guo C, Yue M (2019). Cullin5 deficiency promotes small-cell lung cancer metastasis by stabilizing integrin β1. J Clin Invest.

[26] Zhang S, Wang W, Wang E, Qiu X (2012). SOCS3 expression is inversely correlated with Pyk2 in non-small cell lung cancer and exogenous SOCS3 inhibits proliferation and invasion of A549 cells. Pathology.

[27] Wang J, Wang Y, Sun D, Bu J, Ren F, Liu B (2017). miR-455-5p promotes cell growth and invasion by targeting SOCO3 in non-small cell lung cancer. Oncotarget.

[28] Hassler C, Cruciat CM, Huang YL, Kuriyama S, Mayor R, Niehrs C (2007). Kremen is required for neural crest induction in Xenopus and promotes LRP6-mediated Wnt signaling. Development.

[29] Li Y, Lu W, King TD, Liu CC, Bijur GN, Bu G (2010). Dkk1 stabilizes Wnt co-receptor LRP6: implication for Wnt ligand-induced LRP6 down-regulation. PLoS ONE.

[30] Akca H, Tani M, Hishida T, Matsumoto S, Yokota J (2006). Activation of the AKT and STAT3 pathways and prolonged survival by a mutant EGFR in human lung cancer cells. Lung Cancer.

[31] Wu M, Zhang P (2020). EGFR-mediated autophagy in tumourigenesis and therapeutic resistance. Cancer Lett.

[32] Liu D, Sheng C, Gao S, Yao C, Li J, Jiang W (2015). SOCS3 Drives Proteasomal Degradation of TBK1 and Negatively Regulates Antiviral Innate Immunity. Mol Cell Biol.

[33] Li X, Grisanti M, Fan W, Asuncion FJ, Tan HL, Dwyer D (2011). Dickkopf-1 regulates bone formation in young growing rodents and upon traumatic injury. J Bone Miner Res.

[34] Mao B, Wu W, Davidson G, Marhold J, Li M, Mechler BM (2002). Kremen proteins are Dickkopf receptors that regulate Wnt/beta-catenin signalling. Nature.

[35] Cselenyi CS, Lee E (2008). Context-dependent activation or inhibition of Wnt-beta-catenin signaling by Kremen. Sci Signal.

[36] Nakamura T, Nakamura T, Matsumoto K (2008). The functions and possible significance of Kremen as the gatekeeper of Wnt signalling in development and pathology. J Cell Mol Med.

[37] Wang SQ, Wang C, Chang LM, Zhou KR, Wang JW, Ke Y (2016). Geridonin and paclitaxel act synergistically to inhibit the proliferation of gastric cancer cells through ROS-mediated regulation of the PTEN/PI3K/Akt pathway. Oncotarget.

[38] Johnson DE, O'Keefe RA, Grandis JR (2018). Targeting the IL-6/JAK/STAT3 signalling axis in cancer. Nat Rev Clin Oncol.

[39] Yu JS, Cui W (2016). Proliferation, survival and metabolism: the role of PI3K/AKT/mTOR signalling in pluripotency and cell fate determination. Development.

[40] Bournazou E, Bromberg J (2013). Targeting the tumor microenvironment: JAK-STAT3 signaling. JAKSTAT.

[41] Tomas A, Futter CE, Eden ER (2014). EGF receptor trafficking: consequences for signaling and cancer. Trends Cell Biol.

[42] Maemondo M, Inoue A, Kobayashi K, Sugawara S, Oizumi S, Isobe H (2010). Gefitinib or chemotherapy for non-small-cell lung cancer with mutated EGFR. N Engl J Med.

[43] Cairns J, Fridley BL, Jenkins GD, Zhuang Y, Yu J, Wang L (2018). Differential roles of ERRFI1 in EGFR and AKT pathway regulation affect cancer proliferation. EMBO Rep.

[44] Tiemin P, Fanzheng M, Peng X, Jihua H, Ruipeng S, Yaliang L (2020). MUC13 promotes intrahepatic cholangiocarcinoma progression via EGFR/PI3K/AKT pathways. J Hepatol.

[45] Song J, Liu Y, Liu F, Zhang L, Li G, Yuan C (2021). The 14-3-3σ protein promotes HCC anoikis resistance by inhibiting EGFR degradation and thereby activating the EGFR-dependent ERK1/2 signaling pathway. Theranostics.

[46] Yue F, Jiang W, Ku AT, Young AIJ, Zhang W, Souto EP (2021). A Wnt-Independent LGR4-EGFR Signaling Axis in Cancer Metastasis. Cancer Res.

[47] Shtiegman K (2007). Defective ubiquitinylation of EGFR mutants of lung cancer confers prolonged signaling. Oncogene.

[48] Huang KY, Kao SH, Wang WL, Chen CY, Hsiao TH, Salunke SB (2016). Small Molecule T315 Promotes Casitas B-Lineage Lymphoma-Dependent Degradation of Epidermal Growth Factor Receptor via Y1045 Autophosphorylation. Am J Respir Crit Care Med.

[49] Croker BA, Kiu H, Nicholson SE (2008). SOCS regulation of the JAK/STAT signalling pathway. Semin Cell Dev Biol.

[50] Kario E, Marmor MD, Adamsky K, Citri A, Amit I, Amariglio N (2005). Suppressors of cytokine signaling 4 and 5 regulate epidermal growth factor receptor signaling. J Biol Chem.

[51] Bullock AN, Rodriguez MC, Debreczeni JE, Songyang Z, Knapp S (2007). Structure of the SOCS4-ElonginB/C complex reveals a distinct SOCS box interface and the molecular basis for SOCS-dependent EGFR degradation. Structure.

[52] Nicholson SE, Metcalf D, Sprigg NS, Columbus R, Walker F, Silva A (2005). Suppressor of cytokine signaling (SOCS)-5 is a potential negative regulator of epidermal growth factor signaling. Proc Natl Acad Sci U S A.

[53] Orabona C, Pallotta MT, Volpi C, Fallarino F, Vacca C, Bianchi R (2008). SOCS3 drives proteasomal degradation of indoleamine 2,3-dioxygenase (IDO) and antagonizes IDO-dependent tolerogenesis. Proc Natl Acad Sci U S A.

