# Supplementary material for: Kremen2 drives the progression of non-small cell lung cancer by preventing SOCS3-mediated degradation of EGFR
Source: J Exp Clin Cancer Res. 2023 Jun 3;42:140. doi: 10.1186/s13046-023-02692-3 (PMC10239115; doi:10.1186/s13046-023-02692-3)
Supplement: Supplementary file 1 — Additional file 1. [file 13046_2023_2692_MOESM1_ESM.pdf]

Supplementary Information

Kremen2 drives the progression of non-small cell lung cancer by preventing SOCS3-mediated degradation of EGFR

Sun, et al

Supplementary Figure S1.

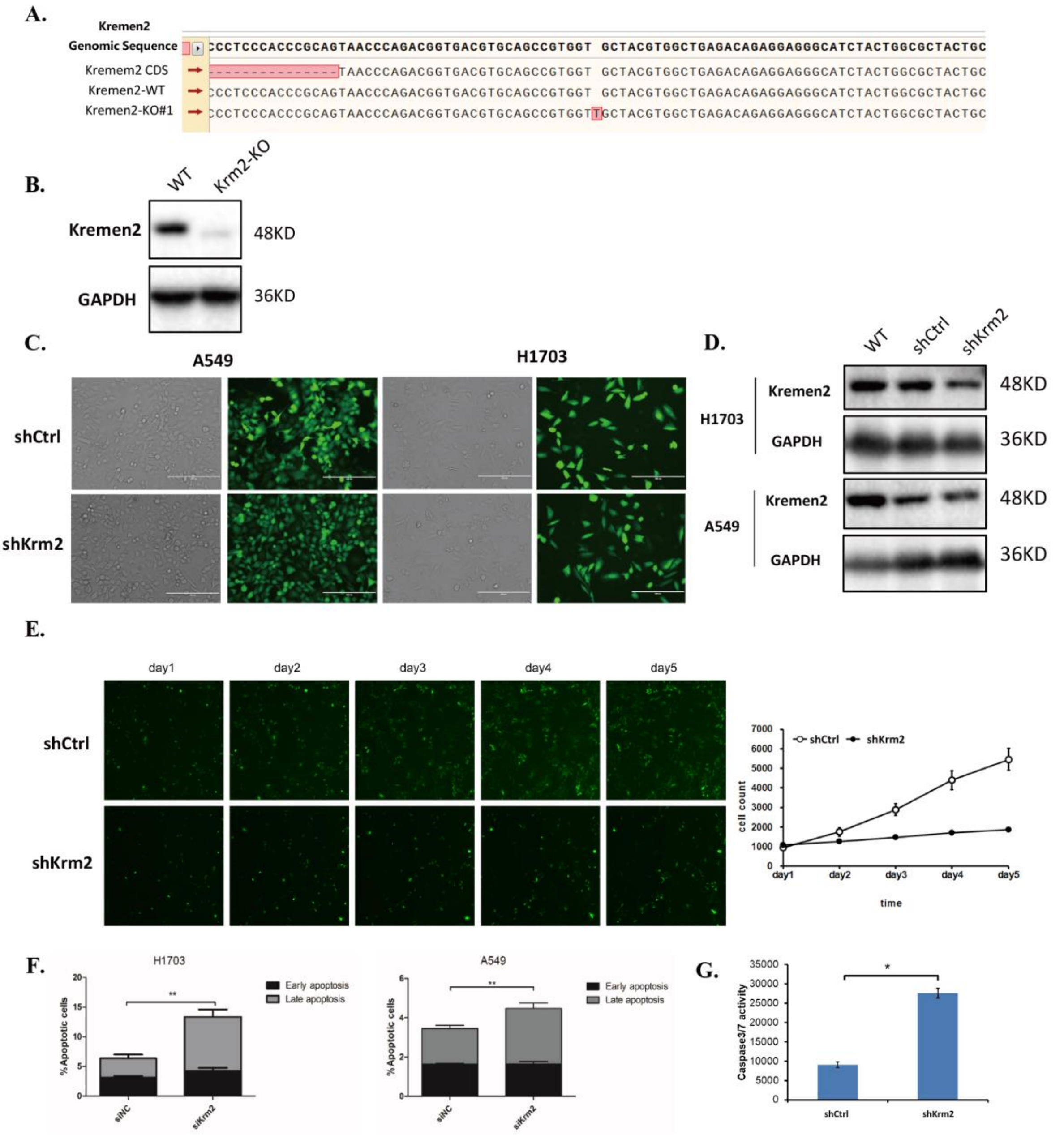

Fig. S1. Construction of the Kremen2 knockout cell line and the effect of Kremen2 on cell proliferation and apoptosis. **A** DNA sequence alignment of Kremen2-knockout cells with the wild-type Kremen2 sequence. The mutant base is inserted between 298 and 299 in the coding sequence (CDS) of Kremen2. **B** Kremen2 knockout in A549 cells was validated by western blot. **C** Lentivirus infection efficiency was monitored through GFP in A549 and H1703 cells. **D** Kremen2 expression was stably knocked down using shRNA. **E** The proliferation ability of shCtrl and shKrm2 cells was determined using the Celigo cell-counting assay in A549 cells. **F** The level of apoptosis of siNC and siKrm2 cells was determined by flow cytometry of H1703 and A549 cells. **G** The activity of Caspase 3/7 was detected in A549 cells treated with shCtrl or shKrm2.

Supplementary Figure S2.

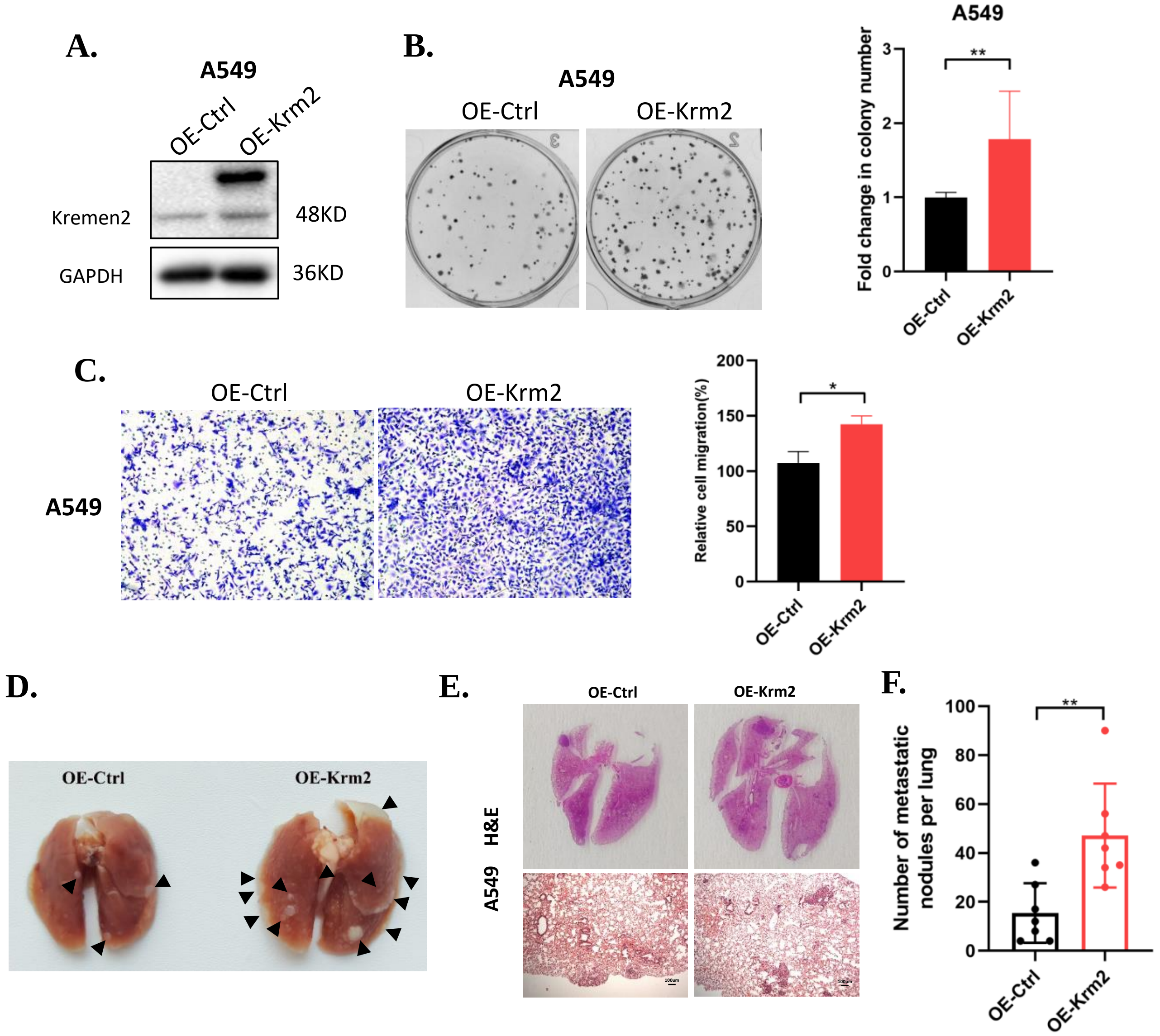

Fig. S2. The effect of Kremen2 overexpression on cell proliferation and migration. **A** The lentivirus-mediated overexpression of Kremen2 (OE-Krm2) in A549 cells was validated by western blot. **B** Colony formation assays were conducted with A549 cells transduced by OE-Ctrl or OE-Krm2 lentiviruses. **C** Transwell assays were carried out with A549 cells transduced by OE-Ctrl or OE-Krm2 lentiviruses. **D-E** Representative image (**D**) and H&E staining (**E**) of the lungs from mice injected with A549-OE-Ctrl cells or A549-OE-Krm2 cells (scale bars: 100 μm). **F** The number of metastatic nodules per lung was measured (n = 7).

Supplementary Figure S3.

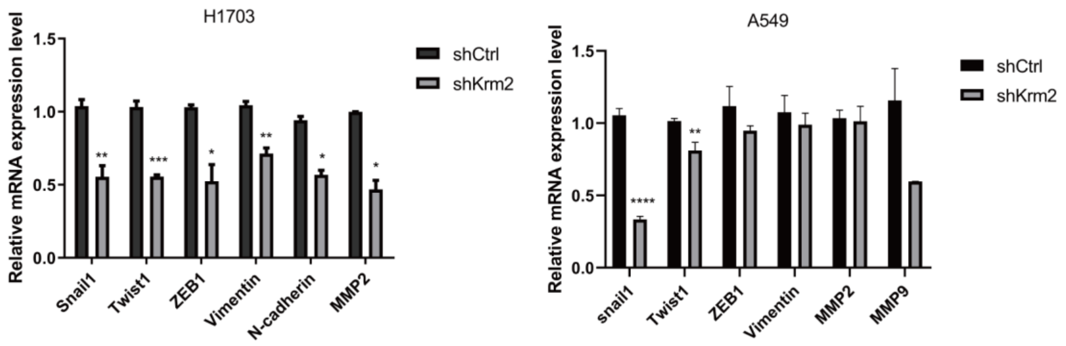

Fig. S3. Relative mRNA levels of EMT-related factors were detected by RT-qPCR in Kremen2-knockdown cells.

Supplementary Figure S4.

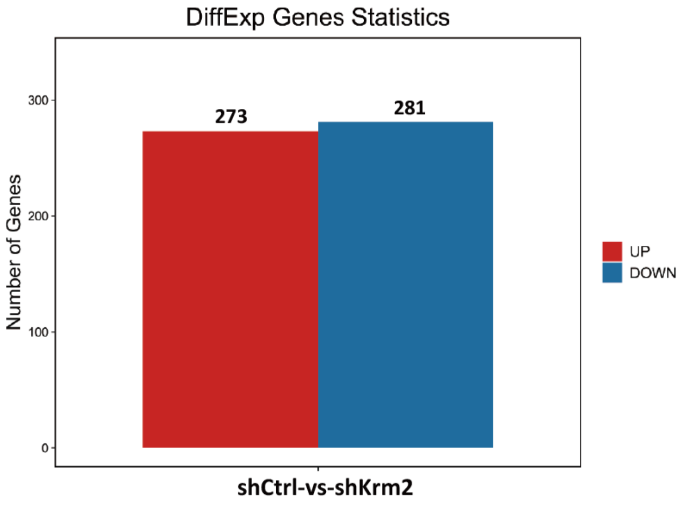

Fig. S4. The differentially expressed genes in H1703 Kremen2-knockdown cells were identified based on fold changes in expression (≥1 or ≤ −1), as shown in the histogram.

Supplementary Figure S5.

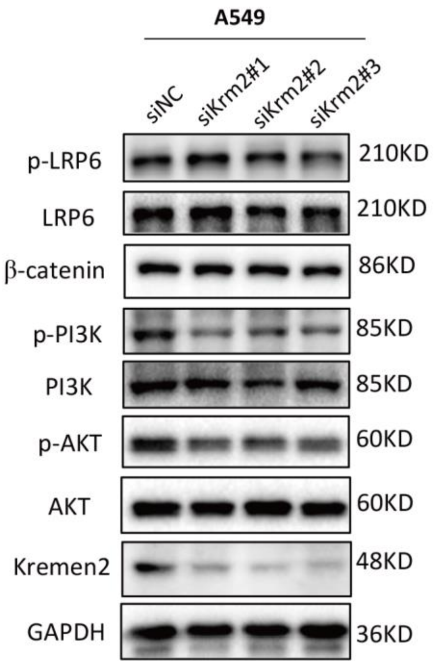

Fig. S5. Protein levels of the Wnt signaling and PI3K/AKT pathway proteins were detected by western blot in A549 cells with Kremen2 knockdown.

Supplementary Figure S6.

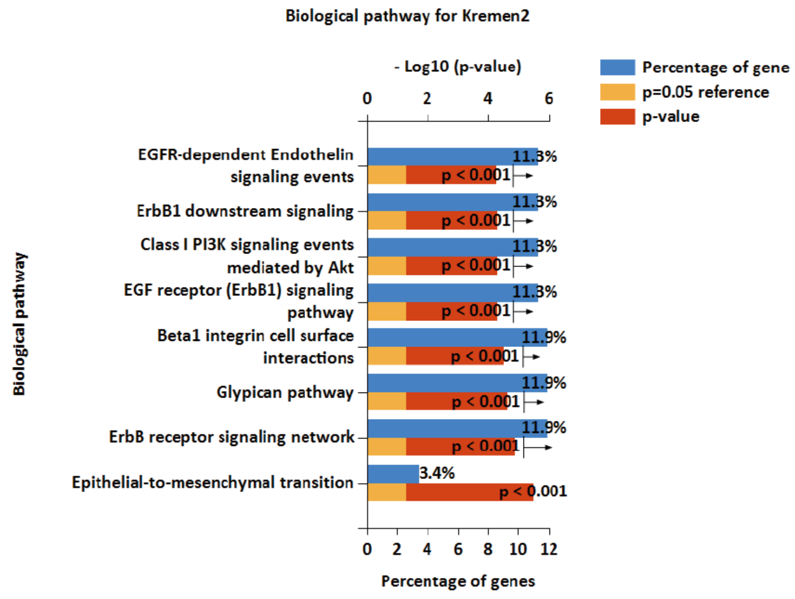

Fig. S6. Bioinformatics analysis of Kremen2-knockdown cells. Data from differentially expressed genes using FunRich software (version 3.1.3) for the functional analysis.

Supplementary Figure S7.

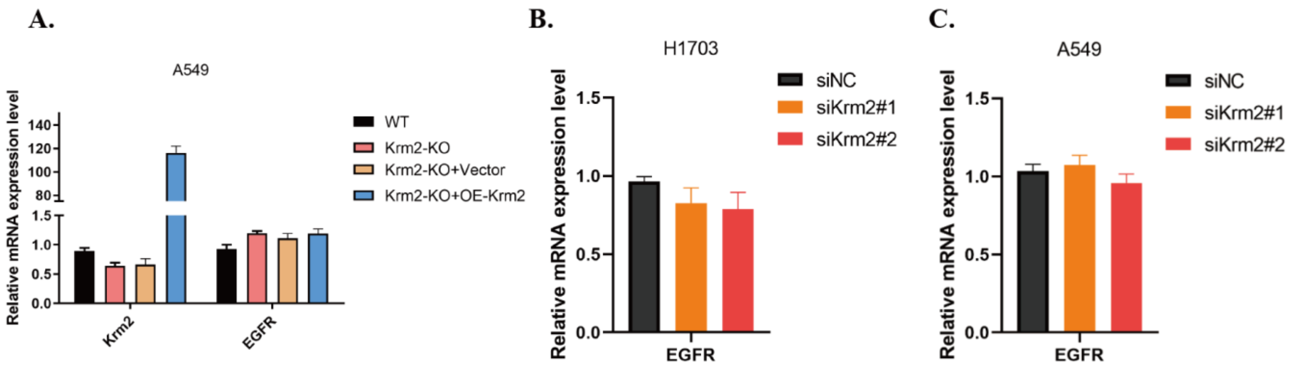

Fig. S7. EGFR mRNA levels in different Kremen2-depleted cells. **A** EGFR mRNA levels in Kremen2 knockout cells. **B** EGFR mRNA levels in Kremen2 knockdown H1703 cells treated with siRNAs. **C** EGFR mRNA levels in Kremen2 knockdown A549 cells treated with siRNAs.

Supplementary Figure S8.

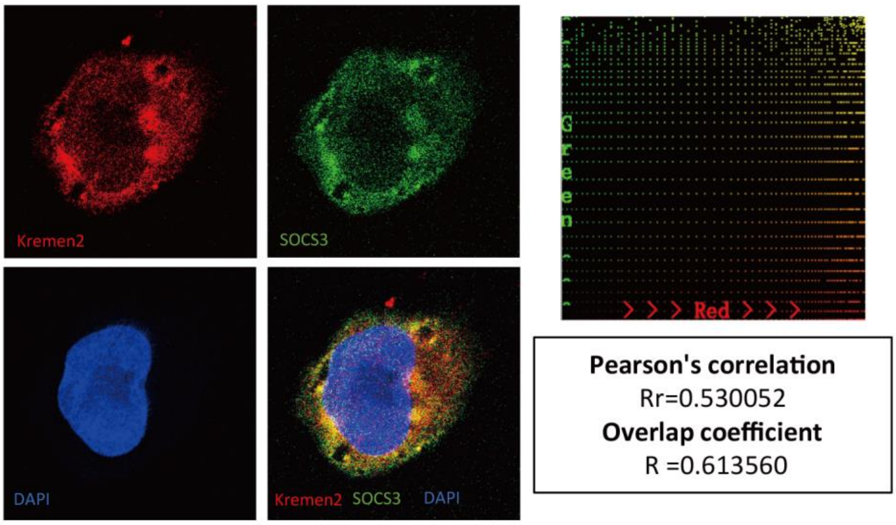

Fig. S8. Co-localization of red (Kremen2) and green (SOCS3) was analyzed by Image-Pro Plus 6.0 in H1703 cells.

Supplementary Figure S9.

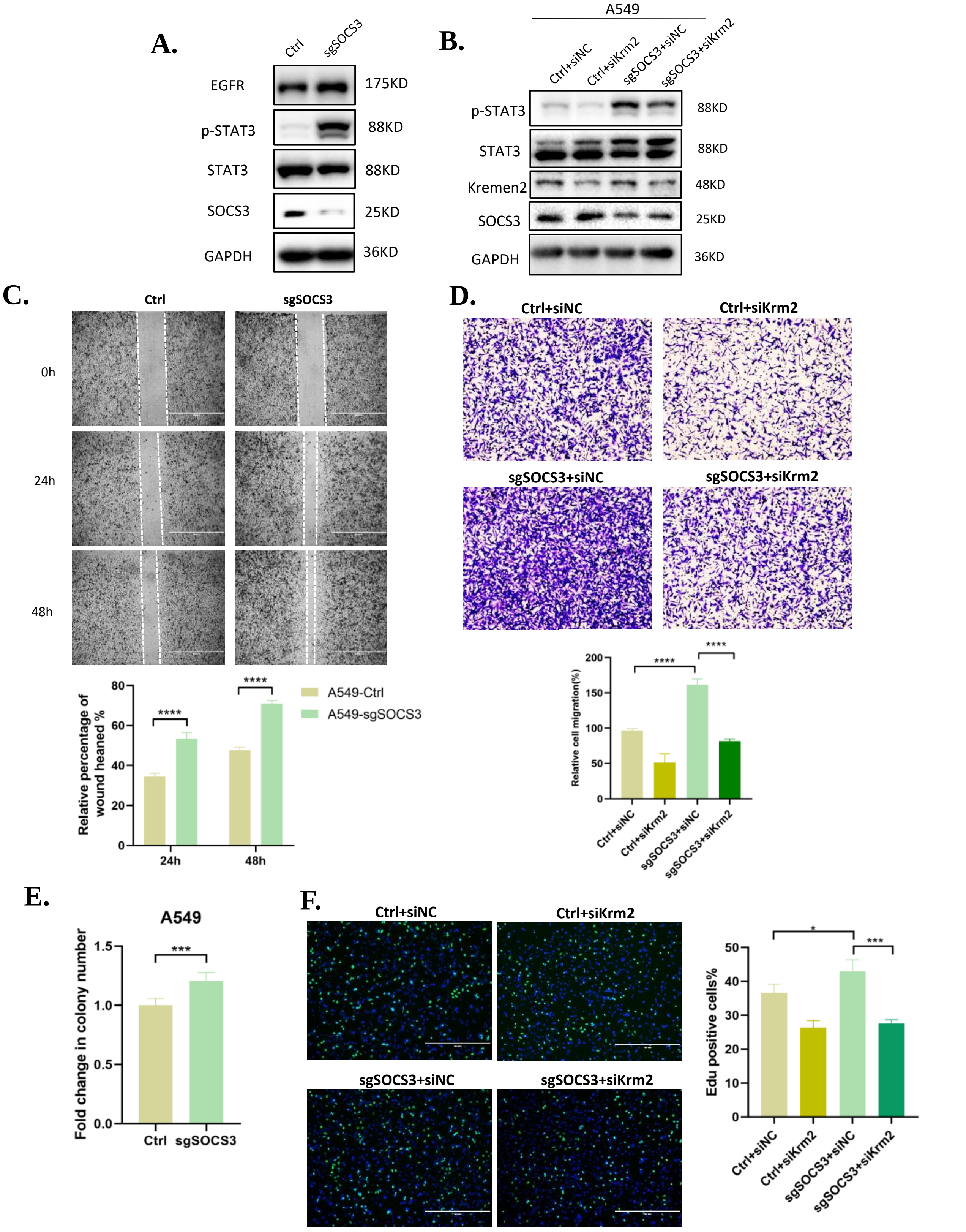

Fig. S9. Silencing of endogenous SOCS3 expression promotes cell proliferation and migration. **A** SOCS3 knockout in A549 cells using CRISPR/Cas9 was validated by western blot. **B** SOCS3 knockout cells were transfected with siKrm2 and indicated proteins were analyzed by IB analysis. **C** Cellular migration was examined by wound healing assay with Ctrl or SOCS3 knockout A549 cells. **D** Transwell migration assays were carried out after SOCS3 knockout A549 cells treated with or without siKrm2. **E** Colony formation assays were conducted using SOCS3 knockout A549 cells. **F** Cell proliferation was detected by EdU assay in SOCS3 knockout A549 cells treated with or without siKrm2.

Supplementary Figure S10.

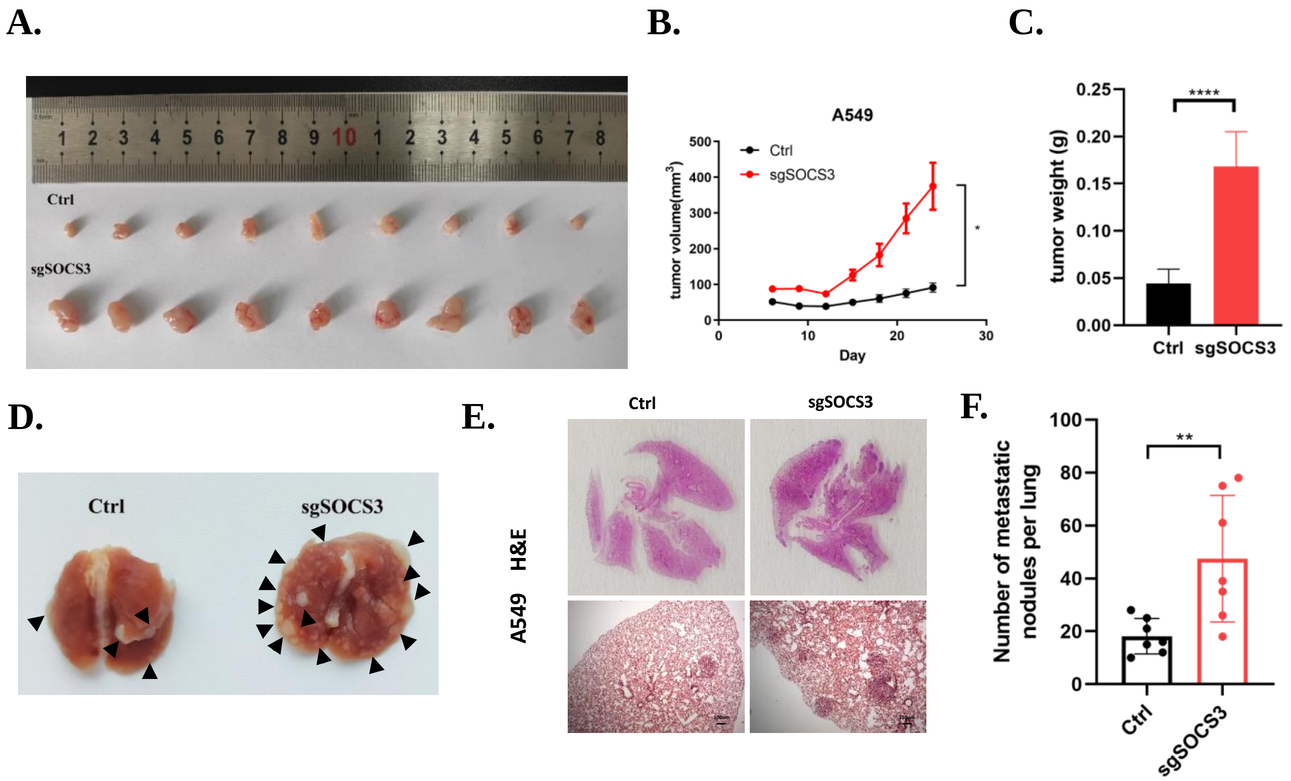

Fig. S10. Knockout of SOCS3 promotes NSCLC cell proliferation and metastasis *in vivo*. **A-C** A subcutaneous xenograft model of nude mice was established using SOCS3 knockout A549 cells (n = 9). Representative xenograft tumor images **(A)**, tumor volume curve (**B**) and final tumor weight (**C**). **D-E** A549-Ctrl cells or A549-sgSOCS3 cells were injected into nude mice to establish lung metastasis model. Representative lung images (**D**) and H&E staining (**E**) of the lungs from mice (scale bars: 100 μm). **F** The number of metastatic nodules per lung was counted (n = 7) .
